# AB569, a Novel, Topical Bactericidal Gel Formulation, Kills Pseudomonas aeruginosa and Promotes Wound Healing in a Murine Model of Burn Wound Infection

**DOI:** 10.1128/IAI.00336-21

**Published:** 2021-10-15

**Authors:** Amanda Barry, Warunya Panmanee, Daniel J. Hassett, Latha Satish

**Affiliations:** a Shriners Hospitals for Children–Cincinnati, Cincinnati, Ohio, USA; b Department of Molecular Genetics, Biochemistry and Microbiology, University of Cincinnati College of Medicine, Cincinnati, Ohio, USA; c Department of Pathology and Laboratory Medicine, University of Cincinnati College of Medicine, Cincinnati, Ohio, USA; University of Pennsylvania

**Keywords:** burn wounds, infections, *Pseudomonas aeruginosa*, bactericidal, AB569, acidified nitrite, EDTA, wound healing, burns

## Abstract

Cutaneous thermal injuries from burns/explosives are a major cause of morbidity and mortality and represent a monumental burden on our current health care system. Injury severity is predominantly due to potentially lethal sepsis caused by multidrug-resistant (MDR) bacteria such as Pseudomonas aeruginosa (MDR-PA). Thus, there is a critical need to develop novel and effective antimicrobials for the (i) prevention, (ii) treatment, and (iii) healing of such wounds that are complicated by MDR-P. aeruginosa and other bacterial infections. AB569 is a novel bactericidal tandem consisting of acidified NaNO_2_ (A-NO_2–_) and Na_2_-EDTA. Here, we first show that AB569 acts synergistically to kill all human burn wound strains of P. aeruginosa *in vitro.* This was found to be due, in part, to the generation of A-NO_2–_-mediated nitric oxide (NO) formation coupled with the metal chelating properties of Na_2_-EDTA. Using a murine scald burn wound model of P. aeruginosa infection, an AB569-Solosite gel formulation eradicated all bacteria. Futher, we also demonstrate enhanced AB569-mediated wound healing by not only accelerating wound contraction, but also by reducing levels of the proinflammatory cytokines interleukin-6 (IL-6) and IL-1β while increasing the levels of anti-inflammatory cytokine, IL-10, and granulocyte-colony-stimulating factor (G-CSF). We also observed better epidermal restoration in AB569-treated wounds. Taken together, we conclude that this study provides solid foundational evidence that AB569 can be used topically to treat highly problematic dermal insults, including wound, burn, blast, and likely, diabetic infections in civilian and military populations, and help relieve the economical burden that MDR organisms have on the global health care system.

## INTRODUCTION

Despite improvements in early treatment, survival following burn injury remains challenging, due largely to sepsis, the leading cause of death in pediatric (1) and adult (2) burn patients. Sepsis is often preceded by infectious complications (3). One of the greatest challenges in treating bacterial infections is their resistance to conventional antibiotics. Multidrug-resistant (MDR) bacteria now account for the bulk of deaths due to sepsis (3), which is the most expensive health care problem in the United States, with a cost of more than $20 billion annually (4). Infection is an even greater cause of death from burn trauma in military personnel than in the general population (5). Pseudomonas aeruginosa is the most frequently cultured source of infection in burn patients (6), accounting for over half of all severe burn infections (7), and is among the major causes of sepsis after burn trauma (8). Within a few days of admission, 14 to 33% of burn wounds are colonized with P. aeruginosa (9, 10). Moreover, infection is the main cause of delayed wound healing in various types of wounds, including burns (11). The Centers for Disease Control and Prevention (CDC) has earmarked P. aeruginosa as a major pathogen and MDR organism (12) responsible for life-threatening infections in critically ill or immunocompromised patients (8). Due to the intrinsically high acquired antibiotic resistance of P. aeruginosa to many, and in some cases all, of the conventional antimicrobial treatments used to date, treatment of burn wound infections caused by P. aeruginosa is both challenging and frustratingly limited, especially since the development of promising new antimicrobial agents has slowed to a trickle (3). Hence, there is a critical and urgent unmet need to develop novel and effective antimicrobials for the treatment and prevention of bacterial burn/blast/wound infections by formidable pathogens such as MDR-P. aeruginosa.

AB569, an innovative, bactericidal combination of acidified nitrite (A-NO_2–_) and Na_2_-EDTA, has been recently shown to have broad-spectrum activity against virtually all pathogenic bacteria (13). Regarding human use, the NaNO_2_ and/or EDTA component(s) of AB569 have separately been proven safe in studies related to the treatment of cyanide poisoning (14), burn wounds (15), cystic fibrosis (CF) lung infection (16), urinary tract infection (17), wound healing (15, 18), chelation therapy (19), and cosmetics (20). Furthermore, both components of AB569 have been reported to increase the efficacy of certain antibiotics that are commonly used to treat a variety of infections (13, 21, 22). Our recent work (13) showed that AB569 has excellent bactericidal activity against all tested Gram-positive (G^+^) and Gram-negative (G^–^) bacteria, including those that are MDR. Importantly, in that study, we also we observed no discernible toxicity of AB569 to human airway (e.g., CF), skin (e.g., burn wounds), or bladder (e.g., urinary tract infections [UTIs]) cells or in a mouse model of P. aeruginosa airway infection and no development of resistance by bacteria cultured *in vitro* (13, 23). However, little is known regarding the potential of the A-NO_2–_ and EDTA combination in the treatment of P. aeruginosa-mediated burn wound infection and in wound healing, a far more clinically simple topical assessment of AB569 efficiacy than complicated airway delivery systems.

Thus, the overall goals of this study were to (i) determine the efficacy of AB569 in reducing or eradicating the P. aeruginosa burden in burn wounds and (ii) promote wound closure and healing. Our interests also included investigating the potential for AB569 in healing uninfected burn wounds. To address these goals, we first developed a water-based gel formulation of AB569 and tested its efficacy on clinical strains of P. aeruginosa isolated from human burn wounds. Strikingly, bacterial killing was observed in all P. aeruginosa strains tested. Furthermore, we successfully established the *in vivo* effectiveness of AB569 application in killing P. aeruginosa in a complex, burn wound infection mouse model without systemic infection and in promoting and enhancing the wound healing process. Taken together, the overall efficacy and improved treatment of P. aeruginosa infections by AB569 are critically important, timely, and highly promising therapeutic discoveries for the treatment of infected burn wounds.

## RESULTS

### AB569 kills P. aeruginosa burn wound clinical isolates *in vitro*.

We first tested the bactericidal activity of AB569 against various clinical strains of P. aeruginosa isolated from burn wound patients along with a bioluminescent strain of wild-type P. aeruginosa strain Xen41 (PA-Xen41) (PAO1 derivative) by synergy measurements using classical checkerboard analysis (24). To determine what concentrations are effective for each chemical independently, MICs or combined fractional inhibitory concentrations (FICs) were assessed. This assay allows for the determination of the FIC index, an indicator of synergy, via the equation FIC_Index_ = (FIC_A_/MIC_A_) + (FIC_B_/MIC_B_). If the result of the two fractions is less than 0.5, the combination is considered synergistic. If the result is between 0.5 and 1.0, the combination can be considered weakly synergistic (13). Otherwise, 1.0 to 4.0 is considered additive, and 4.0+ is antagonistic. Interestingly, our results showed a weak synergistic killing effect (FIC < 1) of the components of AB569 on all P. aeruginosa clinical isolates and PA-Xen41 (see Table S1 in the supplemental material).

### Vehicle development of an AB569 delivery system for the treatment of P. aeruginosa-infected burn wounds.

It is well established in previous studies that A-NO_2–_ possesses the potential to kill a myriad of MDR G^+^ and G^–^ pathogens (13, 25–27). The bactericidal tandem AB569 (A-NO_2_– combined with Na_2_-EDTA) was not recognized as an antimicrobial agent in its own right until just recently (13). EDTA is generally regarded as a “potentiator” of other antimicrobial agents (22, 28). EDTA also perturbs bacterial membranes in part by chelating Ca^2+^ and Mg^2+^ in the lipopolysaccharide (LPS) layer of G^–^ bacteria, thereby structurally taxing the outer membrane by allowing significant LPS release (29). AB569 has been shown *in vitro* to be bactericidal, as it kills both G^–^ and G^+^ organisms and is nontoxic to human airway, skin, and bladder epithelia (13). Our recent findings demonstrated that AB569 can cause a massive, catastrophic downregulation of P. aeruginosa genes involved in vital cellular functions, including the biosynthesis of DNA, RNA, protein, and respiration (ATP synthase, succinate dehydrogenase, cytochrome *c* oxidase), as well as anaerobic metabolism and type III secretion [13]). This highly promising discovery warranted further *in vivo* investigation of the role of AB569 in the potential eradication of P. aeruginosa in burn wound infections. However, to deliver AB569 topically to burn wounds, a suitable delivery vehicle was first necessary.

Initially, AB569 was compounded using a water-based gel (Solosite, here, SS) at a pH between 6 and 6.5 (necessary for optimal bactericidal activity). The efficacy of this formulation was tested *in vitro* using an overnight culture of PA-Xen41 diluted in LB medium (pH 6.5) along with SS at a 1:100 dilution. SS is routinely used by our institution (Shriners Hospitals for Children–Cincinnati [SHC-C]) for the treatment of burn patients to maintain wound moisture and is the water-based gel of choice for patients inflicted with deep partial-thickness burns and when tendons are exposed. Interestingly, the gel in combination with 2 mM EDTA exhibited significant killing of the PA-Xen41, although some luminescence was observed after 48 h. In contrast, there were no bioluminescent signals with SS in combination with either 30 mM NaNO_2_ or 2 mM EDTA + 30 mM NaNO_2_, indicating complete killing of P. aeruginosa (Fig. S1A and B).

The efficacy of the AB569 gel formulation was next tested on different clinical strains of P. aeruginosa isolated from human burn patients that demonstrated various sensitivities and resistances to antibiotics using a broth-based killing assay. We found that AB569 at a concentration of 2 mM EDTA and 30 mM NaNO_2_ killed P. aeruginosa, irrespective of its antibiotic sensitivity or resistance (Fig. S1C). Interestingly, 2 mM EDTA alone showed efficient killing of clinical P. aeruginosa strains. Taken together, these data suggest that the topical SS gel does not interfere with the overall effectiveness of the bactericidal components of AB569.

### Pharmacokinetic analysis of nitric oxide (NO) generated by AB569.

Critical pharmacokinetic studies were performed next to ascertain that the gel does not interfere with the release of antimicrobial nitric oxide (NO) from A-NO_2–_. Yoon et al. (30) previously showed that acidification of NO_2–_ results in formation of nitrous acid (HNO_2_). However, HNO_2_ is unstable toward disproportionation and favors further and sustained production of NO. To test this postulate using the AB569-SS formulation, NO polarographic measurements were performed using an ISO-NOP electrode linked to an Apollo 4000 detector. The production of NO from the combination of bacterial culture medium (L-broth), 10% SS gel, and AB569 (1 mM EDTA, 15 mM A-NO_2–_) was observed for ∼8 h at a maximum concentration of bactericidal levels of NO (57 nM; Fig. S2), indicating that the gel does not interfere with the production of NO by AB569.

**FIG 1:**
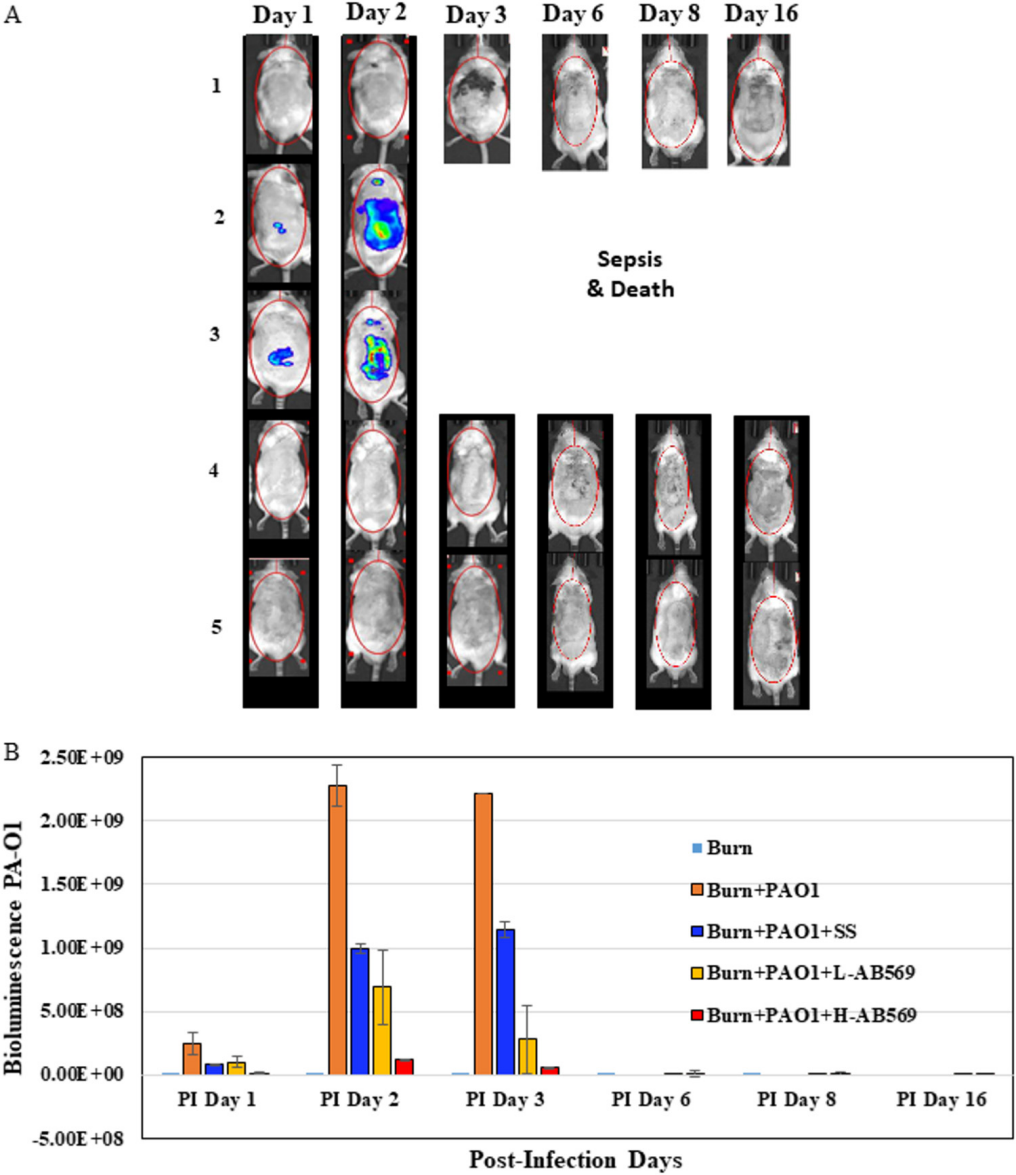
AB569 effectively prevents the growth of PAO1 in burn wounds. (A) Representative IVIS images of burn wounds infected and treated with high dose (33 mM EDTA + A-NO_2–_ formulated in 1% SS gel) and low dose (33 mM EDTA + 500 mM A-NO_2–_ in 1% SS gel) of AB569 shown on PBD 2, 3, 4, 7, and 9. (B) Quantification of photon intensities of bacterial burden showing significant reduction in photon intensities of PAO1 XEN41 in animals treated with low and high AB569. (1) Burn alone (*n* = 2); (2) burn + PAO1 (*n* = 7); (3) burn + PAO1 + SS (*n* = 7); (4) burn + PAO1 + L-AB569 (*n* = 12); (5) burn + PAO1 + H-AB569 (*n* = 23). L-AB569, low AB569; H-AB569, high AB569; SS, Solosite. Student’s *t* test showed significance comparing burn + PAO1 to burn + PAO1 + L-AB569 and burn + PAO1 + H-AB569 on postinfection days 3 and 6. **, *P* < 0.001; ***, *P* < 0.0001.

**Effect of AB569 on** **P. aeruginosa** **using a scald burn wound infection model.** To determine the efficacy of AB569 in the killing of P. aeruginosa in an infected wound, a scald burn wound model (31) was utilized with few modifications. The infected scald burn wound model was established in CD-1 mice. Then, 24 h postburn, the wounds were infected by topical inoculation with bioluminescent PA-Xen41. The establishment of P. aeruginosa colonization and infection on the skin burns were determined using the IVIS imaging system. Our findings clearly indicated the presence of a burn wound infection in the skin between 24 and 48 h. After several iterations of compounding using the low and high doses of AB569 with SS gel as specified above, we found that the topical application of the AB569-gel formulation in two different doses, 30 mM NaNO_2_ and 2 mM EDTA (low dose, L-AB569) and 500 mM NaNO_2_ and 33 mM EDTA (high dose, H-AB569), pH 6.0 to 6.5, eliminated the infection, with the high dose having a more robust effect (Fig. 1A and B). Treatments were initiated immediately after burn injury, which was followed by two administrations on the day of infection and 24 h postinfection. Treatments were then continued with single applications until postburn day (PBD) 7. Our results suggest that AB569 prevented the initial attachment and growth of P. aeruginosa on the infected burn wounds, consistent with recent results demonstrating active dispersion from preformed, mature biofilms (13). Above or below the aforementioned concentrations, the formulation lost some viscosity and was deemed unsuitable for topical application. From this point, we next focused on the L- and H-AB569 modalities.

### AB569 positively impacts the wound healing process.

**FIG 2:**
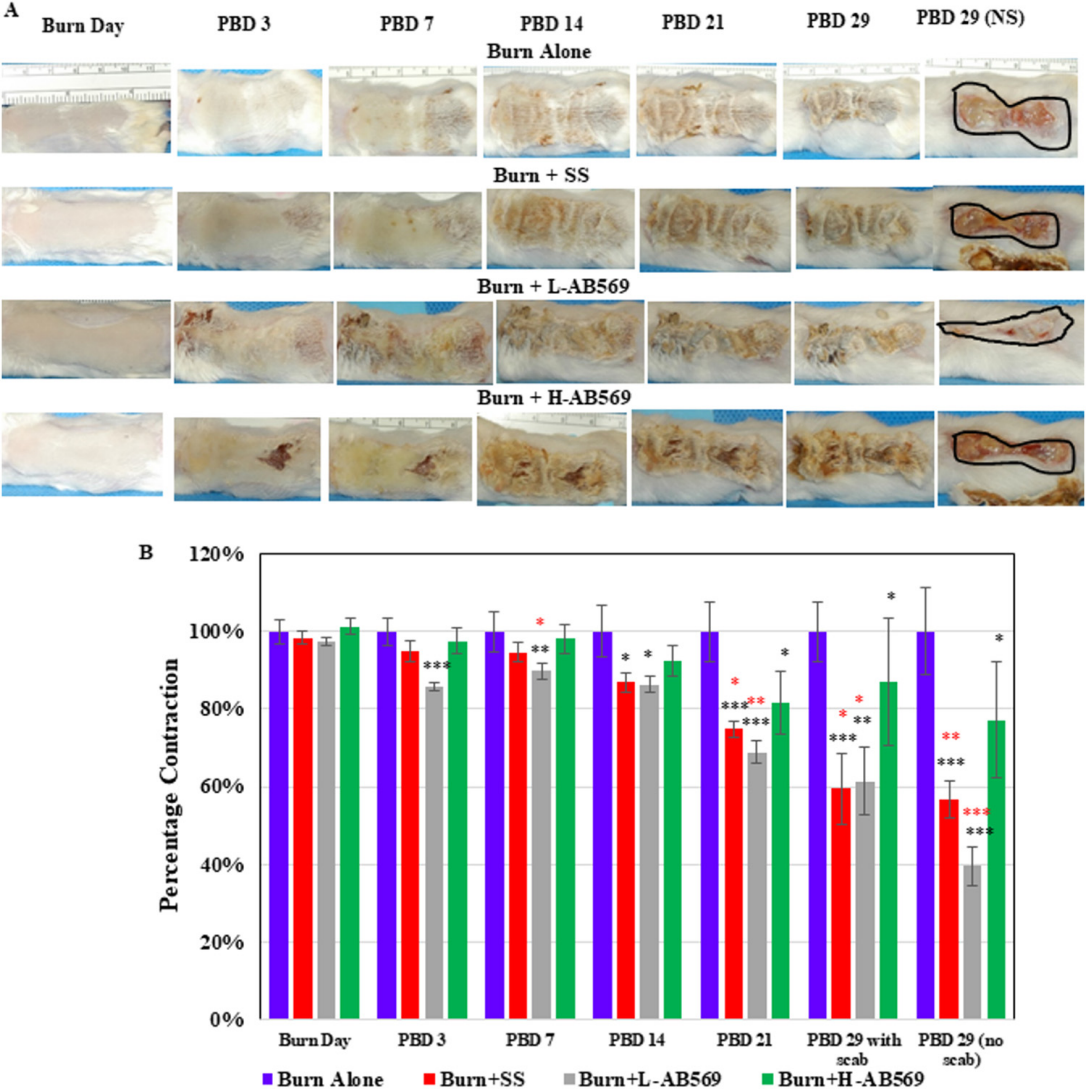
AB569 enhances wound closure in uninfected burn wounds. (A) Gross images of the wounds were captured, and analysis of wound closure was performed using NIH ImageJ. Wounds were photographed weekly using a standard digital camera. (B) Rate of wound closure in uninfected treated burn wounds measured by Image J from burn day to PBD 29. The data are normalized to burn for each post burn day. Student’s *t* test compared the black (*) *P* values to burn day and the red (*) *P* values to high AB569 on respective days. (burn, *n* = 20; H-AB569, *n* = 23; SS, *n* = 22; L-AB569, *n* = 20;). NS, no scab.

Bacterial wound infection triggers a robust inflammatory response, tissue damage, and healing retardation. Previous studies have not addressed the utility of A-NO_2–_ and/or EDTA in the healing process in burn wounds. To assess the impact of AB569 on the localized response of burn wound tissue without P. aeruginosa infection, our initial studies focused on grossly examining wound contraction in various treatment groups compared to untreated burn wounds (Fig. 2A). Our results indicate that by postburn day 29 with no scab (NS), SS alone (57 ± 4.8%) and SS plus L-AB569-treated wounds (40% ± 5%) triggered significant wound contraction. Interestingly, the wound contracting ability of the SS plus H-AB569 (77% ± 4%) was less than SS alone. It should be noted, however, that the L-AB569 formulated with SS significantly enhanced wound contraction relative to SS alone by greater than 17% (*P* value of 0.013; Fig. 2B). Thus, for the first time, we have demonstrated that SS alone has wound healing benefits due likely to its hydrating of the extracellular matrix (allantoin) and antimicrobial/bacteriostatic (methylparaben, propylparaben, benzyl alcohol) properties. However, when SS was admixed with NaNO_2_ and EDTA, the effect was dramatically enhanced by more rapid wound closure and increased mouse survival. Another clinically relevant finding of these experiments was the enhancement of wound closure observed in infected wounds treated with H-AB569, while infected and infected + SS mice all died PBD 3 (Fig. S3A). Furthermore, a significant enhancement in wound healing was observed in P. aeruginosa wounds treated with H-AB569 (71% ± 15%) on PBD 29, showing a clear reduction in wound size (Fig. S3B).

### AB569 significantly improves the survival of burn wound animals infected with P. aeruginosa.

**FIG 3:**
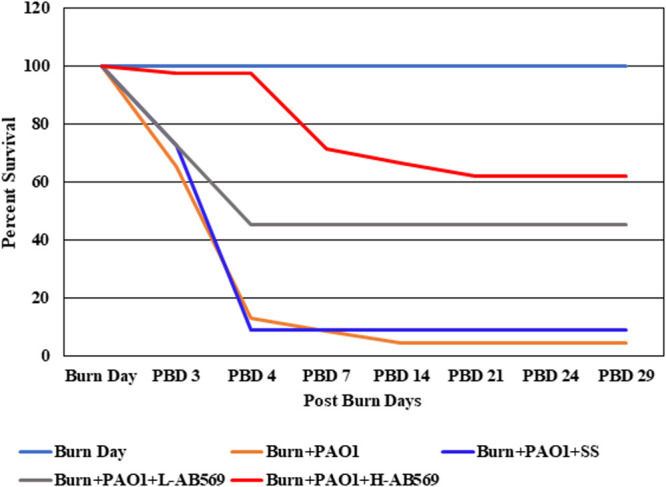
Survival rates of P. aeruginosa-infected animals increased after AB569 treatment. The survival rates of infected wounds from burn day to PBD 24 in all the treatment groups (total no. of animals, *n* = 109).

Full-thickness scald burn injured animals that were uninfected, uninfected and treated with SS, and uninfected and treated with L-AB569 formulated with SS showed a 100% survival rate (Fig. S4). Uninfected burn wounds with H-AB569 formulated with SS showed 91% survival on PBD 7, 82% survival rate on PBD 14, and 74% survival on PBD 29. H-AB569 also enhanced wound closure/contraction. In some cases, we noted that there could be an increased risk of death upon multiple treatments. However, animals infected with P. aeruginosa and treated with L-AB569 formulated with SS showed a 46% survival rate from PBDs 4 to 29. In contrast, infected animals treated with H-AB569 in SS exhibited a 97% survival rate on PBD 4, and 62% survival on PBD 29. Untreated burn wound infected animals and those treated with SS alone succummed to death except for one animal within 72 to 96 h (Fig. 3; see Tables 2 and 3). Thus, our remaining focus was only on H-AB569 in P. aeruginosa-infected wounds, as the survival rate was higher than that for infected wounds treated with L-AB569.

### AB569 influences body weight after burn wound infection.

Since body weight is an index of an animal’s overall health after burn injury, animal weights were recorded daily postburn. A significant reduction in the body weights of the uninfected burn wound animals was observed on PBD 3, but the animals regained their weight by PBD 21 (Fig. S5A). In contrast, animals with infected burn wounds either treated with H-AB569 formulated in SS or treated with SS alone showed a significant weight loss on PBD 3, and the latter died within this period. In contrast, animals with H-AB569-treated infected wounds showed a progressive increase in body weight from PBD 24 to PBD 29 (Fig. S5B).

### AB569 protects infected burn wound animals by altering spleen function.

To ensure that AB569 treatment of the murine burn wounds did not elicit any systemic side effects, the relevant sepsis-related organs harvested from sacrificed animals were carefully screened using several important health parameters. First, there were no significant changes in the size, shape, and weight of the liver, lungs, and kidneys. However, significant changes were observed in both the size and weight of the spleen. In both untreated and treated uninfected burn wounds, the spleens of the animals were enlarged and increased in weight progressively from PBD 3 to day 29 (Fig. S6A). Conversely, P. aeruginosa-infected animals treated with H-AB569 which survived infection showed significant increases in spleen weights and enlargement from PBD 3 to PBD 29 (Fig. S6B). These results suggest that an increase in spleen size may indirectly protect the animal from trauma and infection, especially given that two important functions of the spleen are microorganism clearance and removal of antigens. This hypothesis requires further investigation. Similarly, when the body weight to spleen ratio was calculated in the infected and uninfected wounds treated or not treated with AB569, a smaller ratio indicated that the animals had a improved likelihood of survival (Fig. S6C and D). Thus, these results suggest that smaller spleen size is correlative with an increased chance of death.

### AB569 significantly alters the mRNA and protein expression levels of IL-6 and IL-10 in burn wounds.

**FIG 4:**
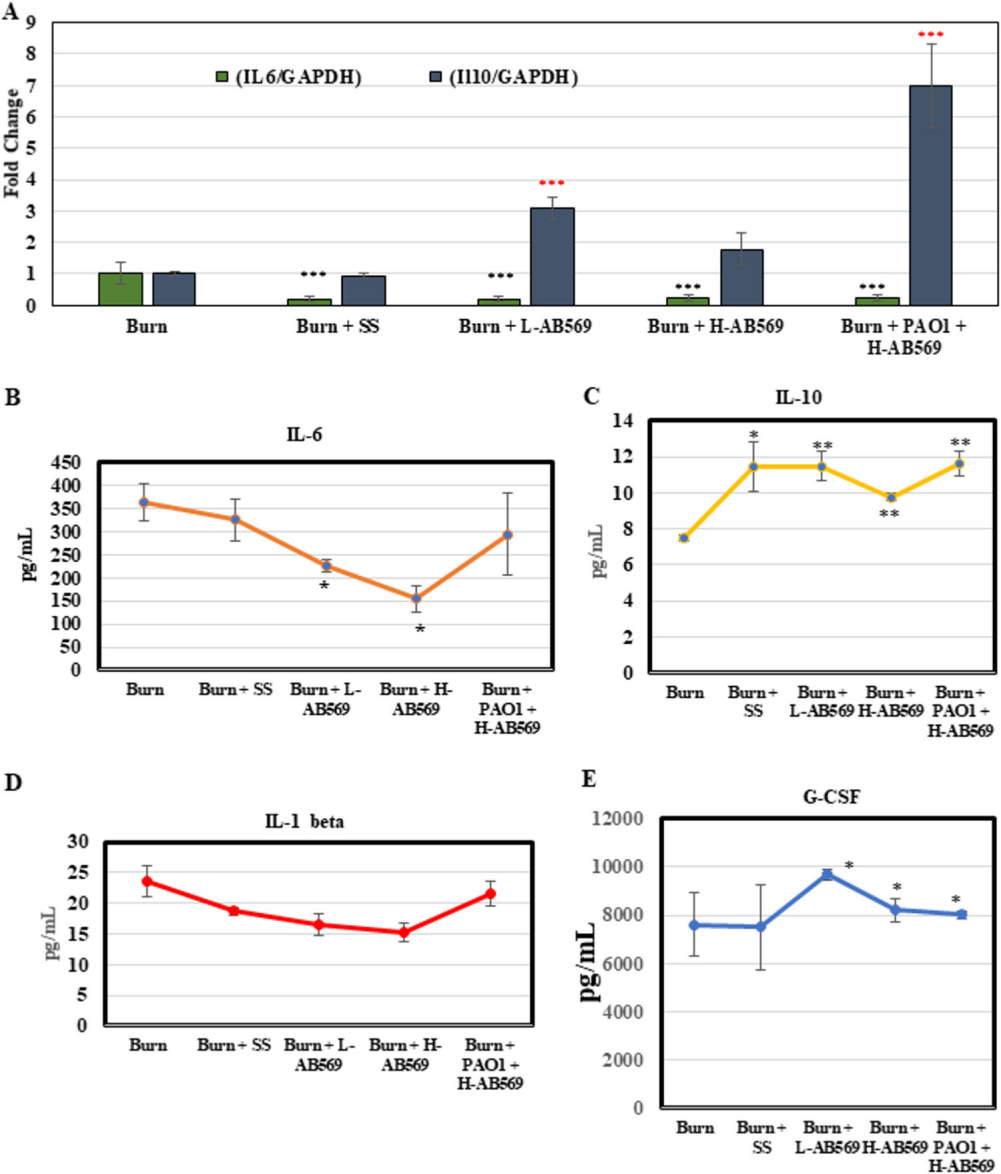
AB569 significantly alters select pro-and anti-inflammatory cytokines in both infected and uninfected burn wounds. (A) Real-time RT-PCR was performed using RNA isolated from treated and untreated burn wound tissue from PBD 29 on IL-6 and IL-10. Experiments were performed in triplicate from RNA isolated from burn (*n* = 6), SS (*n* = 10), L-AB569 (*n* = 8), H-AB569 (*n* = 7), and PAO1 + H-AB569 (*n* = 9). Data are presented as the fold change expression compared to burn alone. Values are mean ± SEM of experimental samples derived from various sample sizes from each group. ***, *P* < 0.01. IL-6, interleukin-6; IL-10, interleukin-10. (B to E) Serum cytokine levels on samples collected on PBD 29. Milliplex Multiplex kits were used to quantify four cytokines (IL-6, IL-10, IL-1β, and G-CSF). Data are plotted as the mean ± SEM for *n* = 4 for all the samples run in triplicate. Statistical analysis was performed using Student’s *t* test with *P* < 0.05 considered statistically significant. PBD; postburn day, SS, Solosite; L-AB569, low AB569; H-AB569, high AB569.

Burn injury elicits excessive inflammation that often lasts for several days. Hence, we were next interested in determining whether the influx of inflammatory cytokines to the wound was altered due to topical administration of AB569 in the days postinjury. Interestingly, mRNA expression of IL-6 was significantly reduced in all of the treatment groups in comparison to the untreated burn wounds (Fig. 4A). Interestingly, SS-treated wounds also exhibited decreased expression of IL-6. In contrast, IL-10 expression was significantly elevated only in uninfected wounds treated with L-AB569 and P. aeruginosa-infected wounds treated with H-AB569 (Fig. 4A). We examined the levels of key serum cytokines by multiplex analysis of serum collected on postburn day 29. We determined that treatment with AB569 substantially reduced the levels of proinflammatory cytokine IL-6 in both L-AB569- and H-AB569-treated uninfected wounds (Fig. 4B). Further, the levels were decreased in the P. aeruginosa-infected wounds treated with H-AB569 relative to burn alone animals. With regard to IL-1β, though a statistical significance was not observed in treated animals, levels were still low compared to burn alone and SS-treated animals (Fig. 4C). One of the key findings was a significant increase in the levels of IL-10 in all the treatment groups, including SS-treated animals (Fig. 4D). Interestingly, levels of G-CSF, an immunomodulatory cytokine, was significantly reduced in uninfected burn wounds treated with L- and H-AB569 (Fig. 4E).

### AB569 alters inflammatory status and collagen expression but did not alter the mRNA expression levels of type I and type III collagens.

**FIG 5:**
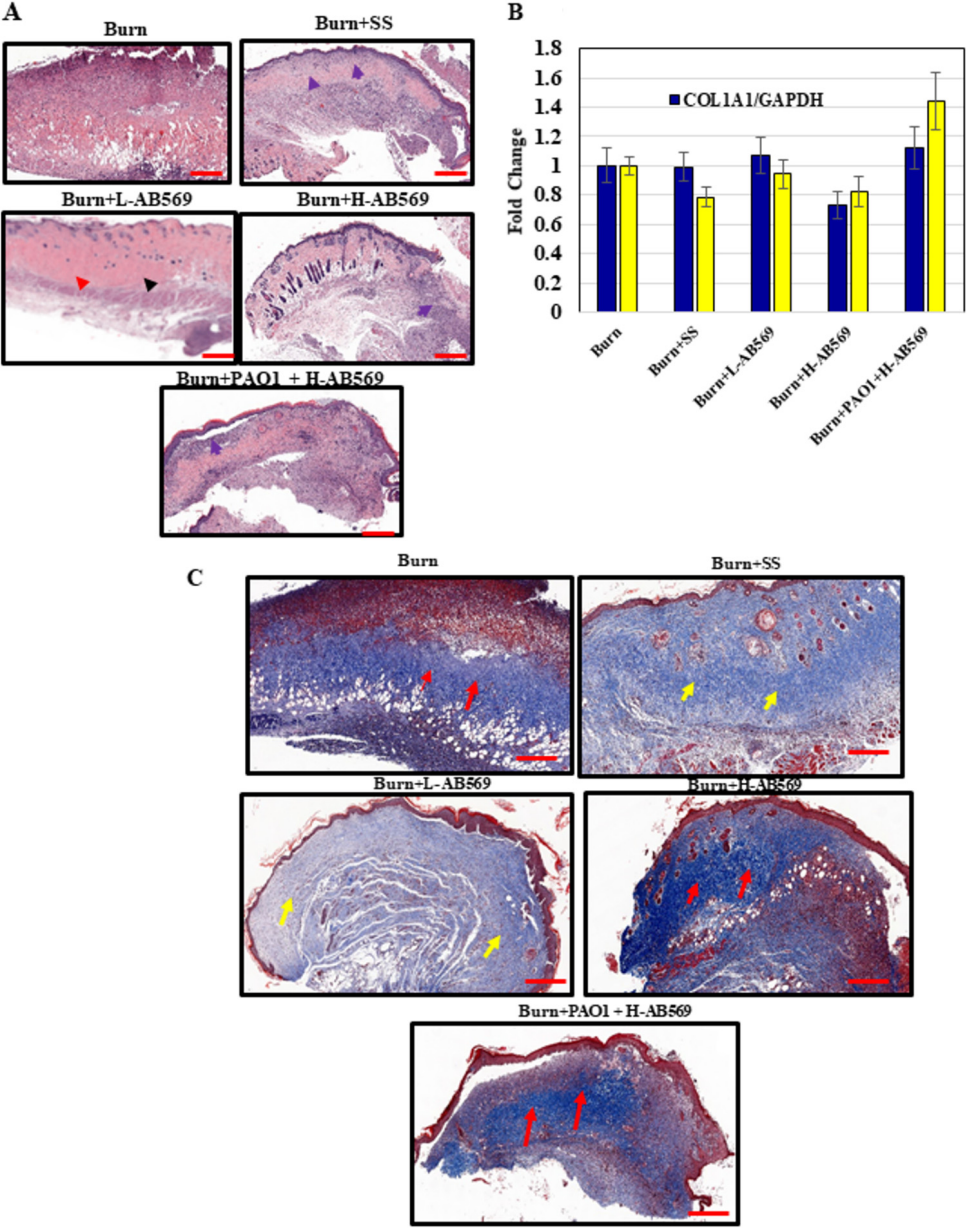
AB569 influences the influx of inflammatory cells and collagen deposition. Real-time RT-PCR and histochemical analyses was performed on tissues harvested on PBD 29, except that the PAO1-infected wound tissues were from PBD 3. (A) Hematoxylin and eosin (H & E) on tissue samples shows significant reduction in the presence of inflammatory cells in burn wound tissues treated with L-AB569 compared to burn-alone tissues. The presence of fibroblasts and dense connective tissue was also noticed in L-AB569-treated tissues (red arrows and black arrows). PAO1-infected wounds treated with H-AB569 significantly decreased the presence of inflammatory cells (blue arrows). Shown here is the representation of images captured from *n* = 4 wounds in different animals per group. Scale bars (red, lower right) are noted in the images. (B) Real-time RT-PCR was performed using RNA isolated from treated and untreated burn wound tissue on type I collagen (COL1A1) and type III collagen (COL3A1). Experiments were performed in triplicate from RNA isolated from burn (*n* = 6), SS (*n* = 10), L-AB569 (*n* = 8), H-AB569 (*n* = 7), and PAO1 + H-AB569 (*n* = 9). Data are presented as the fold change expression compared to burn alone. Values are the mean ± SEM of experimental samples derived from various sample sizes from each group. No statistical significance difference was observed in analyzing COL1A1 and COL3A1 expression levels. Experiments were performed in triplicate from RNA isolated from burn (*n* = 6), SS (*n* = 10), (L-AB569 (*n* = 8), H-AB569 (*n* = 7), and PAO1 + H-AB569 (*n* = 9). (C) Masson’s trichrome stain revealed less collagen deposition in the L-AB569-treated group (yellow arrows) than in all the other treatments (red arrows). Shown here is the representation of images captured from *n* = 4 wounds in different animals per group. PBD, post burn day; SS, Solosite; L-AB569, low AB569; H-AB569, high AB569.

Gross images of hematoxylin and eosin (H&E)-stained sections showed significant differences in burn wounds treated with L-AB569. There was an increased prevalence of mature fibroblasts and organized collagen deposition in the L-AB569 (arrows) treated group in comparison to the untreated group with less inflammatory cells (Fig. 5A). H&E stained wound sections from both control and treated groups were evaluated at 4 weeks postburn by pathology via a semiquantitative histological assessment using several parameters that included inflammation, fibrin exudate, presence of fibroblasts, collagen deposition, and capillary proliferation (Table 1). Wounds in the AB569 cohort demonstrated better overall wound healing, and all wounds completely reepithelialized. Wounds in all groups revealed the presence of scab. Beneath the scab, however, there was complete reepithealization in wounds treated with H- and L-AB569. Interestingly, SS-treated wounds also showed complete reepithelialization, suggesting that wound occlusion helps in rapid epithelial coverage. The cumulative histopatholgic score of skin wounds with different treatments showed similar trends between the control and treatment groups, with a slightly lower score observed in wounds treated with L-AB569. At the mRNA level, we did not observe any significant differences in either type I or type III collagens in uninfected wounds treated with both L- and H-AB569 (Fig. 5B). Overall, a slight increase in type III collagen levels was seen in P. aeruginosa-infected wounds treated with H-AB569 which was not statistically significant (Fig. 5B). The Masson’s trichrome stain used to evaluate the collagen content (Fig. 5C) indicated a higher deposition in wounds treated with H-AB569 compared to L-AB569- and SS-treated wounds. Of note, thin collagen fibrils with more organized collagen structure were observed in L-AB569-treated wounds in comparison to the P. aeruginosa plus H-AB569- and H-AB569-treated groups.

**TABLE 1:** Histopathological score of burn wounds in different treatments

Histological differences seen on H&E-stained sections	Burn alone	Solosite	Low AB569	High AB569	PA + high AB569
Acute polymorphonuclear leukocyte inflammation (score 0–4 per wound); 0 = no inflammation; 1 = minimal inflammation; 2 = mild; 3 = moderate; 4 = marked inflammation	4	4	2	3	3
Fibroblasts and capillary proliferation (score 0–3 per wound); 0 = no fibroblasts and blood vessels/capillaries; 1 = few fibroblasts with few capillaries; 2 = intermediate presence of fibroblasts and presence of capillaries; 3 = mature fibroblasts and several blood vessels	1	2	3	2	3
Amt of collagen deposition between fibroblasts (score 0–4 per wound); 0 = normal large amt; 1 = marked; 2 = moderate; 3 = mild; 4 = minimal amt	1	1	4	2	1
Total scoring of the wounds (a higher score signifies better healing outcome)	6	7	9	7	7

**TABLE 2:** Survival rates from burn day to postburn day 29 in all the treatment groups (*n* = 87)

Treatment group	No. of animals surviving on:							
	Burn day	PBD 3	PBD 4	PBD 7	PBD 14	PBD 21	PBD 24	PBD 29
Burn	22	22	22	22	22	22	22	22
SS	22	22	22	22	22	22	22	22
L-AB569	20	20	20	20	20	20	20	20
H-AB569	23	22	21	21	19	17	17	17

**TABLE 3:** The number of animals in each treatment throughout the experiment

Treatment group	No. of animals on:						
	Burn day	PBD 3	PBD 4	PBD 7	PBD 14	PBD 21	PBD 24
Burn	22	22	22	22	22	22	22
P. aeruginosa + H-AB569	42	41	41	30	28	26	26
P. aeruginosa + L-AB569	11	8	5	5	5	5	5
P. aeruginosa + SS	11	8	1	1	1	1	1
P. aeruginosa	23	15	3	2	1	1	1

### AB569 treatment promotes better epidermal restoration.

**FIG 6:**
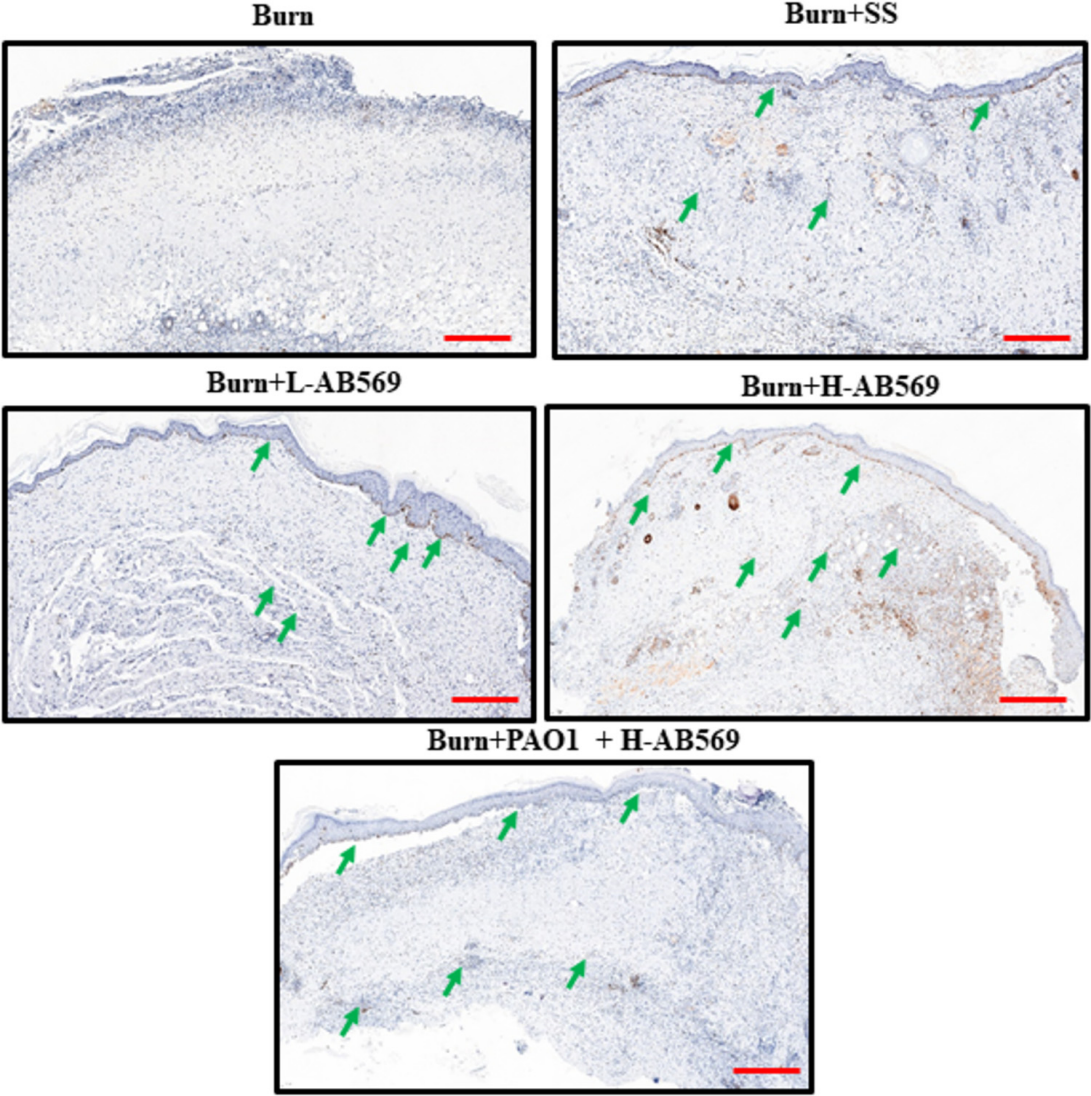
AB569 promotes reepithelialization. Burn wound sections from control and various treatment groups harvested on PBD 29 were stained with Ki67, a marker for proliferating cell nuclei. Representative images of wounds from *n* = 4 wounds in all the different groups stained with Ki67 (brown; green arrows) are shown. Scale bars are noted in the images (red bars, lower right). Complete epithelial coverage was noted in all the treatment groups in comparison to burn alone and PAO1-infected wounds. PBD, postburn day; SS, Solosite; L-AB569, low AB569; H-AB569, high AB569.

The morphological findings in H&E-stained sections show that in addition to complete wound closure, AB569-treated wounds also showed better epidermal restoration. We stained the wound sections with Ki67 to determine the rate of epithelial proliferation. Our data show a complete reepithelialization in infected and uninfected wounds treated either with L- or H-AB569 (Fig. 6). The positive staining of Ki67 observed in the epidermis was significantly higher in wounds treated with L-AB569, whereas in the H-AB569-treated group, the proliferating cells were higher in both epidermis and dermis (Fig. 6). In P. aeruginosa-infected animals, nonspecific staining of Ki67 was observed throughout the tissues, and P. aeruginosa-infected and H-AB569-treated animals showed significant staining in the epidermis, and some staining was also observed in the dermis.

## DISCUSSION

### Burn wound infections.

Infection is the main cause of delayed wound healing in various types of wounds, including burns (32). Burn infection also increases the risk of sepsis and, upon healing, significant scarring (33). The complex microenvironment of burn wounds is an ideal propagative niche suited for various types of infectious microbes to grow and establish themselves as highly refractory, antibiotic-resistant biofilm infections. Burn patients are especially prone to ventilator-associated pneumonia and other wound infections, and the major pathogen implicated in such infections is P. aeruginosa (34). Multiple studies over the past decade have shown that 42 to 65% of deaths in burn victims are attributable to infectious sequelae (35–37). In addition, burn patients with infections have more than twice the mortality of uninfected patients (38). In this regard, if the wound bed is prepared in such a manner that bacterial colonization followed by infection could be mitigated by prophylactic treatment with a bactericidal agent, this would truly be highly beneficial. If such an agent could also hasten wound closure, this property offers an additional therapeutic boon.

### AB569, a powerful antimicrobial and wound-healing agent.

In this study, AB569 (a patented combination of sodium nitrite and Na_2_-EDTA) was compounded using a hydrogel-based vehicle, SS, and tested for its efficacy in not only eliminating P. aeruginosa infection, but also hastening and improving the process of wound healing. Several features of AB569 contributed to its overall efficacy on both of the aforementioned fronts. First, A-NO_2–_-derived NO is a well-known antimicrobial agent (25, 30, 39, 40). Another study showed that NO and A-NO_2–_ offered a novel therapy for the control of methicillin-resistant Staphylococcus aureus (MRSA) in wounds (41). NO is also a component of the human innate immune response involved in the orchestration of wound healing (42, 43). Wound-derived NO critically influences macrophage, fibroblast, and keratinocyte behavior within the intercellular communication network during repair (44). A-NO_2–_ cream, an effective means of topically delivering time- and pH-dependent release of NO, also improved wound healing (18). Second, the EDTA component of AB569 has been shown to disperse and kill P. aeruginosa biofilms (45) and diminish thermal injury progression in a rat model of brass comb burn (15), as well as acting synergistically with multiple antibiotics (46). Furthermore, the application of Livionex lotion (15), which, like AB569, contains Na_2_-EDTA as a metal chelator and methyl sulfonyl methane as a permeability enhancer, reduces the accumulation of reactive aldehydes and protection of aldehyde dehydrogenase enzymes (15), suggesting a benefit of its use in the prevention of burn wound progression.

Our comprehensive study revealed five key findings: (i) AB569 was effectively formulated as a gel, and its components did not impede the release of bactericidal and wound healing levels of NO from A-NO_2–_, as well as the Gram-negative membrane permeabilizing and perturbing properties of EDTA (47), (ii) prophylactic application of AB569 gel likely prevented the colonization and establishment of P. aeruginosa infection in burn wounds, (iii) SS application alone promoted the wound contraction of uninfected burn wounds, (iv) AB569 along with SS hastened the wound healing process more than SS alone, and (v) AB569 treatment modulates the wound bed by altering the expression of inflammatory cytokines.

### AB569, the inflammatory response, and prophylaxis of infection.

Inflammation plays an integral role in the healing of burn wounds, as it influences the sequalae of events necessary for success of this vital process. Prolonged inflammation leads to poor scarring outcomes, resulting in hypertrophic scar formation (2). However, our results clearly point to two different strategies for treatment. After following burn wounds for a period of 30 days to determine the effect of AB569 on uninfected wounds, a dramatic and significant reduction in wound size was noted from postburn day 3 in uninfected wounds treated with L-AB569 (Fig. 2A and B). Surprisingly, H-AB569 did not hinder wound closure in uninfected wounds, but a significant difference from the untreated wounds was observed from postburn day 21 (Fig. 2A and B). In contrast, in infected animals, H-AB569-treated infected wounds not only eliminated the P. aeruginosa infection but enhanced wound closure (Fig. S3C and D), while such a significant difference in wound closure was not observed in L-AB569-treated infected wounds (data not shown). One reason may be that infected wounds required a higher concentration of H-AB569 to clear infection and thereby also provide beneficial attributes for wound closure, which requires further investigation. Significantly, animals treated with AB569 formed a scab covering the underlying healing tissue similar to a cap. The scab formation potentially prevented the dehydration of the healing skin underneath, to protect from infections and to prevent any entry of contaminants from the external environment. Scab formation can also be attributed to the efficient wound contraction seen in treated groups. Histopathological findings show that there was no significant difference in the influx of inflammatory cells between the treated groups in comparison to untreated burn wounds (data not shown) early (postburn day 4) during the wound healing process. However, analysis of postburn wound tissue harvested on PBD 30 showed less influx of inflammatory cells in the AB569-treated group and improved collagen content and organization. One other significant finding from these studies is that the vehicle, SS-treated wounds, showed significant improvement in wound closure and in the pattern of collagen deposition and complete reepithelialization. These findings reiterate that keeping wounds moist provides better healing outcomes. Further mechanistic insights to determine the signaling process through which infection is cleared and healing is achieved are warranted. Based on these findings, our plans are to investigate the efficacy of AB569 on clinical isolates of additional infectious organisms, including two prominent ESKAPE pathogens, S. aureus MRSA and Acinetobacter baumannii. Intriguingly, we showed that AB569-treated wounds decreased the expression of the proinflammatory cytokines IL-6 and IL-1β and increased the expression of the anti-inflammatory cytokine IL-10 and the immunomodulatory cytokine G-CSF on PBD 29, indicating that inflammatory response is not exacerbated in the healing wounds (Fig. 4B to E). G-CSF, a hematopoietic cytokine and potent stem cell mobilization agent, has been shown to play a central role as a regulator of the “genomic storm” driving divergent innate and adaptive immune responses after traumatic injury (48). In the present study, we noted an increased expression of G-CSF in both infected and uninfected wounds which may have positively driven faster clearing of the infection and wound healing, which requires further investigation. The decreased expression of IL-6 and IL-1β in nontreated wounds suggest that these two cytokines can work in concert in prolonging the inflammatory response and thereby triggering the wounds to be chronic and nonhealing. Collectively, our results suggest that L-AB569 may be useful for prophylaxis of burn wound infection, while H-AB569 removes the infection while still enhancing wound closure.

### Future directions.

In conclusion, we have developed a novel, nontoxic bactericidal drug formulation, AB569, for the treatment of burns and other skin infections. AB569 represents a unique agent that has the potential to mitigate infection and accelerate the process of wound healing in burns and other infectious settings. The concentrations of AB569 were not only bactericidal against P. aeruginosa *in vitro* and during burn infection, but also dramatically enhanced the process of wound healing. Given these findings, these studies have dramatic implications for global health, especially in burned patients. Our data also suggest that the AB569 formulations can easily be modified to treat highly problematic infections involving MDR bacteria. Finally, our next plan is to focus on the next logical step to move this technology forward, including animal toxicology and phase I human safety studies.

## MATERIALS AND METHODS

### Strains, media, and growth conditions.

All clinical isolates used in this study were obtained from the microbiology department of Shriners Hospitals for Children–Cincinnati. Bioluminescent PA-Xen41, derived from parental strain PAO1, was purchased from PerkinElmer (Waltham, MA). Luria broth (LB) medium was composed of 10 g tryptone, 5 g yeast extract, and 5 g NaCl (and an additional 15 g Bacto-agar for LB agar) per liter (all chemicals were from Fisher Scientific). Tryptic soy broth (TSB; Becton, Dickinson) and TSB plus 1.5% agar (TSA) were prepared according to the manufacturer’s instructions. All strains listed in Table S1 were grown with appropriate medium plates or broth.

### Checkerboard assays for MIC and FIC determination.

First, 96-well polystyrene plates were filled with 100 μl of LB, pH 6.5, and 10% SS hydrogel (Smith and Nephew, London, UK). Row A was filled with 100 μl of a 4× stock of EDTA (16 mM), and column 10 was filled with 4× NaNO_2_ stock (256 mM), bringing both to a 2× concentration. Next, 2-fold serial dilutions were performed such that a concentration gradient of each was created. Column 12 was filled with medium to represent a negative control. An overnight culture of bacteria was adjusted to an optical density at 600 nm (OD_600_) of 0.5 in LB medium. This was then diluted 1:1,000 into fresh medium, which was then added to the checkerboard plate (columns 1 to 11). This dilution was selected, as it harbored ∼5 × 10^5^ CFU/ml. Column 11 was the positive control. Plates were incubated at 37°C. The cell turbidity was determined using a 96-well plate reader after a 24-h inoculation. The threshold used for a positive cutoff to calculate MICs and fractional inhibitory concentration (FICs) was 0.001 after blank (medium) subtraction.

### Broth-based killing assay.

Bioluminescent PA-Xen41 was grown overnight in LB medium at 37°C with shaking. Overnight cultures were diluted 100-fold into fresh LBN (LB-1% KNO_3_) 6.5 medium, and 5-ml aliquots were transferred to culture tubes. Final concentrations of 30 mM NaNO_2_ and/or 2 mM EDTA were added to each tube. Cells were grown aerobically at 37°C (49). Samples were taken daily for 48 h while the cells were still in the anaerobic chamber. Samples were serially diluted in phosphate-buffered saline (PBS), pH 7.4, and a 10-μl aliquot from each dilution was placed on an LB agar plate and grown aerobically overnight at 37°C. CFU were enumerated the next morning and converted to CFU/ml after multiplying by the dilution factor.

### Full-thickness scald burn wounds.

Male and female CD-1 mice aged 8 to 10 weeks, 27 to 40 g (obtained from Charles River Laboratories, Inc., Wilmington, MA), were housed singly after creation of burn wounds. This study was approved by the University of Cincinnati Institutional Animal Care and Use Committee (protocol no. 17-06-02-01). On the day of burn injury, animals were administered with buprenorphine SR (1 mg/kg) 1 h before the creation of burns. Animals were anesthetized using 4% inhaled isoflurane in oxygen, and a full-thickness, well-demarcated scald burn was created by placing a shaved mouse in a template exposing 28% of the dorsal surface, followed by immersion in a 92.3°C water bath for 9 s. The mouse was then carefully removed, with great care taken not to scratch their backs. The template used was a 60-ml Kendall Luer Lock syringe (catalog [cat.] no. 1186000777; Tyco/Healthcare) with a latex-free tip cap made of polypropylene. Immediately after burn injury, animals were resuscitated by subcutaneous administration of 1.5 ml of 0.9% saline and were placed on a 42°C heating pad to recover.

### Pseudomonas aeruginosa infection.

Bioluminescent PA-Xen41 (derived from parental strain PAO1; PerkinElmer, Waltham, MA) was grown overnight in 5 ml of tryptic soy broth at 37°C with shaking for 16 to 18 h. On PBD 1, mice were anesthetized with 4% isoflurane, and 200 μl of 2 × 10^4^ bioluminescent PAO1 was topically inoculated on the wound using an inoculating loop.

### Preparation of AB569 gel formulation.

Solosite (here, SS; Smith & Nephew, London, UK), a water-based gel, was used as a delivery vehicle for AB569. The density of SS is approximately that of water, 1 g/ml, and therefore, the volume of gel can be determined by mass (i.e., 1 g of SS [Medline Industries, Northfield, IL; REF 449600) is ∼1 ml). Two different concentrations of AB569 were used for the *in vivo* experiments: (ii) 2 mM EDTA and 30 mM NaNO_2_ (L-AB569) and (ii) 33 mM EDTA and 500 mM NaNO_2_ (H-AB569). The desired concentration of EDTA and NaNO_2_ was added to 1 ml SS, the pH was adjusted to 6.0 to 6.5 using 1N HCl, and the pH was confirmed using pH test strips. The ingredients of AB569 were mixed with SS at the time they were applied to the wound. The entire 1 ml of the gel formulation was applied to each wound. Strain PAO1-infected and uninfected burn wounds were either treated or not treated with an L- or H-AB569 SS gel formulation and with SS alone.

The mouse wounds were inoculated with 2e^4^ PAO1 on postburn day 1 and treated with AB569 two times a day on postburn days 1 and 2. Thereafter, wounds were treated once on PBDs 3, 4, and 7. Treatment on PBDs 5 and 6 were based on the bacterial counts seen from IVIS imaging. The establishment of PAO1 colonization and infection on the burns were determined using the IVIS imaging system on PBDs 2 to 4. Gross wound images were taken on postburn days 3, 7, 14, 21, and 29.

### Bioluminescent imaging.

All living animals underwent IVIS imaging (Caliper Life Sciences, Waltham, MA) of their wounds on PBDs 2, 3, 4, and 6 to monitor the growth of bioluminescent P. aeruginosa. All animals were anesthetized with inhaled 2% isoflurane for imaging (exposure time of 2 min). The IVIS camera was maintained at standard settings, as follows: imaging mode, luminescent; exposure time, auto; binning, medium; F/stop, 1; field of view, D. Once compiled, images were compared quantitatively for relative increase/decrease in bioluminescence using Xenogen Living Image software.

### Tissue harvesting.

Animals were sacrificed on approximately PBD 29. If the animals exhibited signs of morbidity and pain, they were sacrificed, usually occurring between 48 and 72 h after infection. The collected wound tissues were stored in 10% formalin for histological analyses, and tissues stored in RNAlater were stored at −80°C for RNA extraction and reverse transcription-quantitative PCR (qRT-PCR) analyses.

### Histological staining.

Histological section analysis was performed on wound tissue to determine the inflammation, epidermal regeneration, and amount of collagen deposition. Thin sections (4 μm) were cut and stained with hematoxylin and eosin (H&E), Mason’s trichrome stain, and Ki67 stain. Stained slides were scanned into digital images with a Thermo Fisher 3DHistech Panoramic DESK scanner with ×40 objective, and images were viewed using CaseViewer and photographed. Sections were stained with H&E to assist in the analysis of infiltration of each cell type, and a subjective score (no, mild, moderate, or severe) was given taking into consideration all live cells within the histological section. The presence of neutrophils, macrophages, and plasma cells was compared between treated and untreated groups. Masson’s trichrome staining was performed to determine the differences qualitatively in the collagen content between the treated and untreated burn wound tissues. All sections were stained with Masson’s trichrome at the same time to eliminate variations in staining.

### Ki67 staining.

Formalin-fixed, paraffin-embedded tissues were cut from representative blocks at a thickness of 4 μm, placed on a charged glass slide, and dried in an oven at 55°C for 3 h. Using the fully automated Leica NOND RXm instrument, the tissue sections were deparaffinized and subjected to heat-induced epitope retrieval using BOND epitope retrieval solution (pH 6.0) for 20 min. Endogenous peroxidase activity was blocked using the Refine detection kit peroxide block solution. Immunohistochemical staining was performed using the *K_i_*-67 recombinant rabbit monoclonal antibody (Thermo Fisher Scientific; [SP6] MA5-14520; 1:100). The staining was visualized using the Leica BOND polymer refine detection kit.

### RNA isolation and quantitative real-time RT-PCR (RT-qPCR).

Approximately 30 to 40 mg of tissue was weighed and homogenized with a bullet blender for 3 min. If not completely homogenized, it was blended for another 3 min and then spun down for 1 min at 13,300 × *g*. After the tissue was homogenized, total RNA was isolated from the burned wound tissue using the RNeasy minikit (Qiagen, Inc., Valencia, CA) following the manufacturer’s instructions. The quantity and quality of RNA were determined by measuring the OD 260/280 ratio using an ND-100 spectrophotometer (Nanodrop Technologies, Inc., Wilmington, DE) and by capillary electrophoresis using an Agilent 2100 BioAnalyzer (Santa Clara, CA). The RNA with an RNA integrity number (RIN) value of >7 was used for RT-qPCR assays.

Total RNA isolated (RNeasy minikit; Qiagen, Inc.) from burn wound tissue treated and untreated with AB569 and treated with SS alone was subjected to RT-qPCR to determine the gene expression levels of collagen types 1 and 3 and MMP-9 genes. QIAshredder columns were used to homogenize samples, followed by running the samples through the genomic DNA (gDNA) eliminator column to remove any genomic DNA present in the sample. cDNA was prepared using the superscript Vilo cDNA kit (Invitrogen/Thermo Fisher Scientific). The TaqMan universal PCR master mix primers used for the following mouse gene products were purchased from Thermo Fisher: GAPDH (Mm05724508_g1), Col3α1 (Mm01254476_m1), Col1α1 (Mm00801666_g1), IL-6 (Mm00446190_m1), and IL-10 (Mm1288386_m1). Real-time PCR was performed using a StepOnePlus real-time PCR system using the following protocol: denaturation at 95°C for 10 min, then 40 cycles of amplification at 95°C for 15 s, followed by annealing and extension at 60°C for 1 min. The comparative 2^–ΔΔ^*^CT^* method (50) was used to determine the expression levels of target genes after normalization to GAPDH expression. The data are presented as fold changes in relative expression levels compared to burn wounds for both treated and nontreated infected and uninfected burn wounds ± the standard error of the mean (SEM).

### Cytokine determinations in serum.

For isolation of serum, whole blood was collected by cardiac puncture, allowed to clot on ice, and centrifuged. Serum samples were frozen at −80°C, and cytokine concentrations were determined using Milliplex Multiplex kits (MilliporeSigma, Darmstadt, Germany) according to the manufacturer’s protocol. Briefly, in a 96-well black plate, 25 μl sample in duplicate was incubated with 25 μl antibody-coated beads overnight at 4°C on a plate shaker. Plates were then washed 2 times using the BioTek 405 TS instrument (BioTek, Winooski, VT), and 25 μl of secondary antibody was added and incubated at room temperature for 1 h with shaking. Finally, 25 μl of streptavidin-R-phycoerythrin (RPE) was added directly to the secondary antibody and incubated for 30 min at room temperature with shaking. Plates were then washed 2 more times, and 150 μl of sheath fluid was added. Plates were shaken for 5 min and then read using Luminex technology on the Milliplex Analyzer (MilliporeSigma). Concentrations were calculated from standard curves using recombinant proteins and expressed in pg/ml. Data analyses were performed by the Research Flow Cytometry Core at Cincinnati Children’s Medical Center.

### Wound area measurements.

Images of the wounds were captured from the day the burn wounds were created. NIH ImageJ software was utilized to determine the extent of wound contraction in AB569-treated, SS-treated, and untreated groups.

### Statistical analysis.

Statistical analysis was performed using Student’s *t* test. A *P* value of <0.05 was considered statistically significant. Student’s paired *t* test was used to compare the differences between the control and experimental groups.
