# Supplementary material for: AB569, a Novel, Topical Bactericidal Gel Formulation, Kills Pseudomonas aeruginosa and Promotes Wound Healing in a Murine Model of Burn Wound Infection
Source: Infect Immun. 2021 Oct 15;89(11):e00336-21. doi: 10.1128/IAI.00336-21 (PMC8519293; doi:10.1128/IAI.00336-21)

Fig.S1A

Barry et al. 2021
Fig. S1

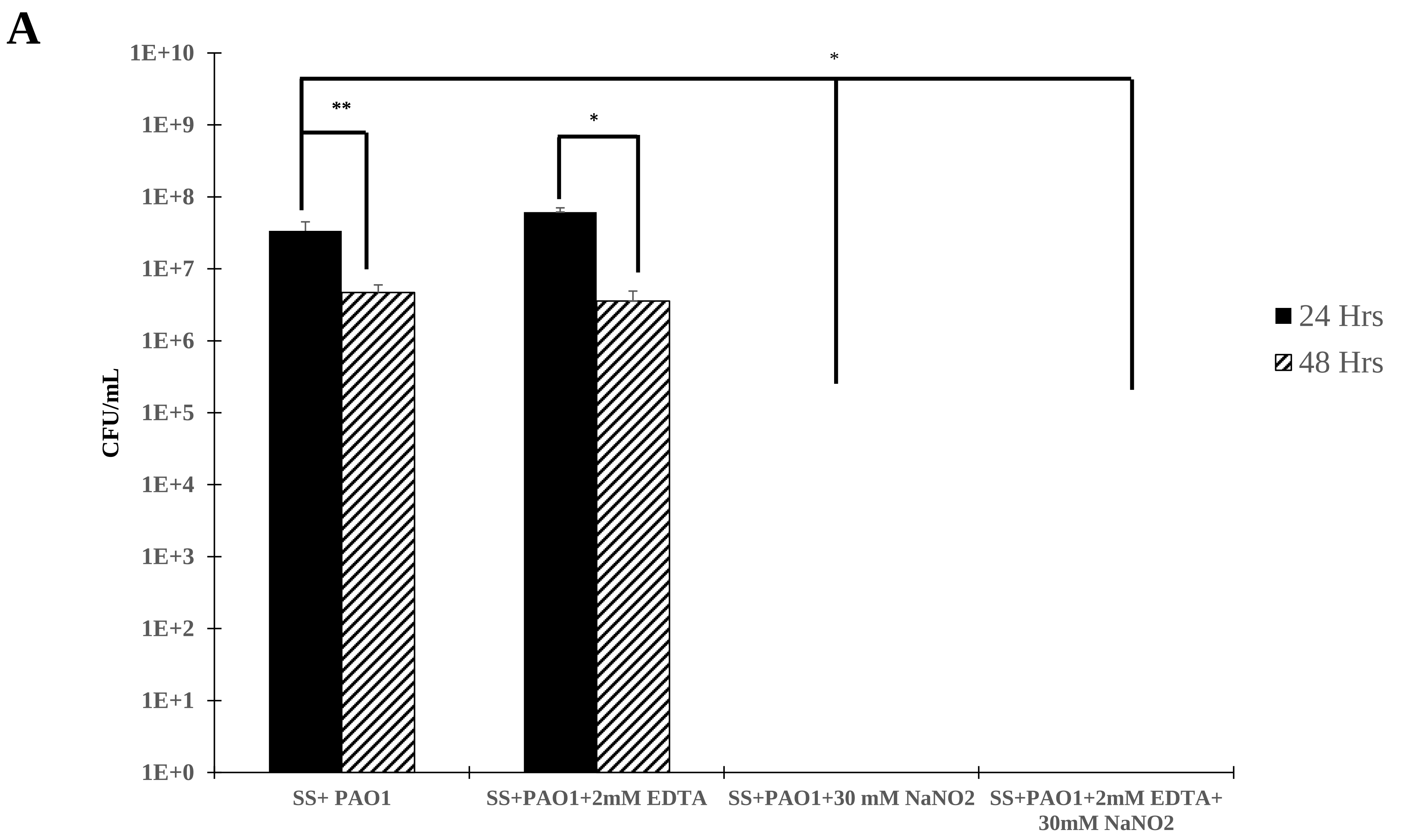

B

48 hour culture Tube 1	48 hour culture Tube 2	48 hour culture Tube 3	48 hour culture Tube 4	48 hour culture Tube 5

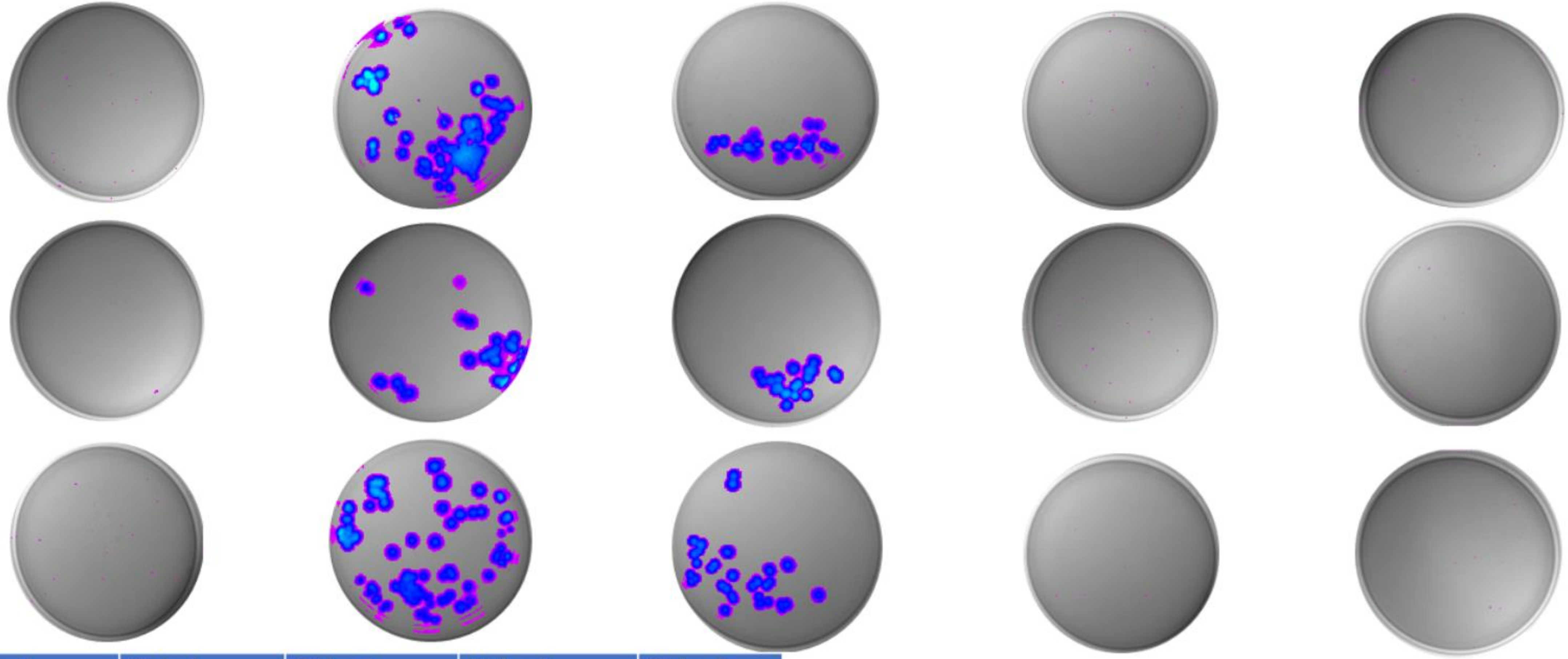

Tube1	Tube2	Tube 3	Tube 4	Tube 5
**LB 6.5 + SS**	LB 6.5 + SS+ PAO1	LB 6.5 + SS +PAO1 + 2mM EDTA	LB 6.5 + SS+ PAO1 + 30 mM NaNO2	LB 6.5 + SS +PAO1 + 2mM EDTA + 30 mM NaNO2

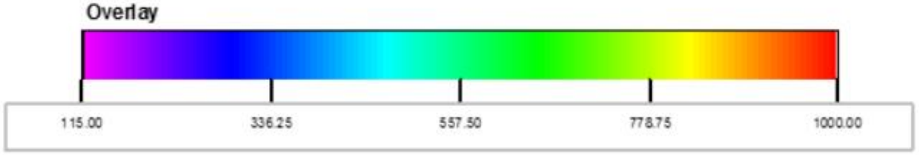

C

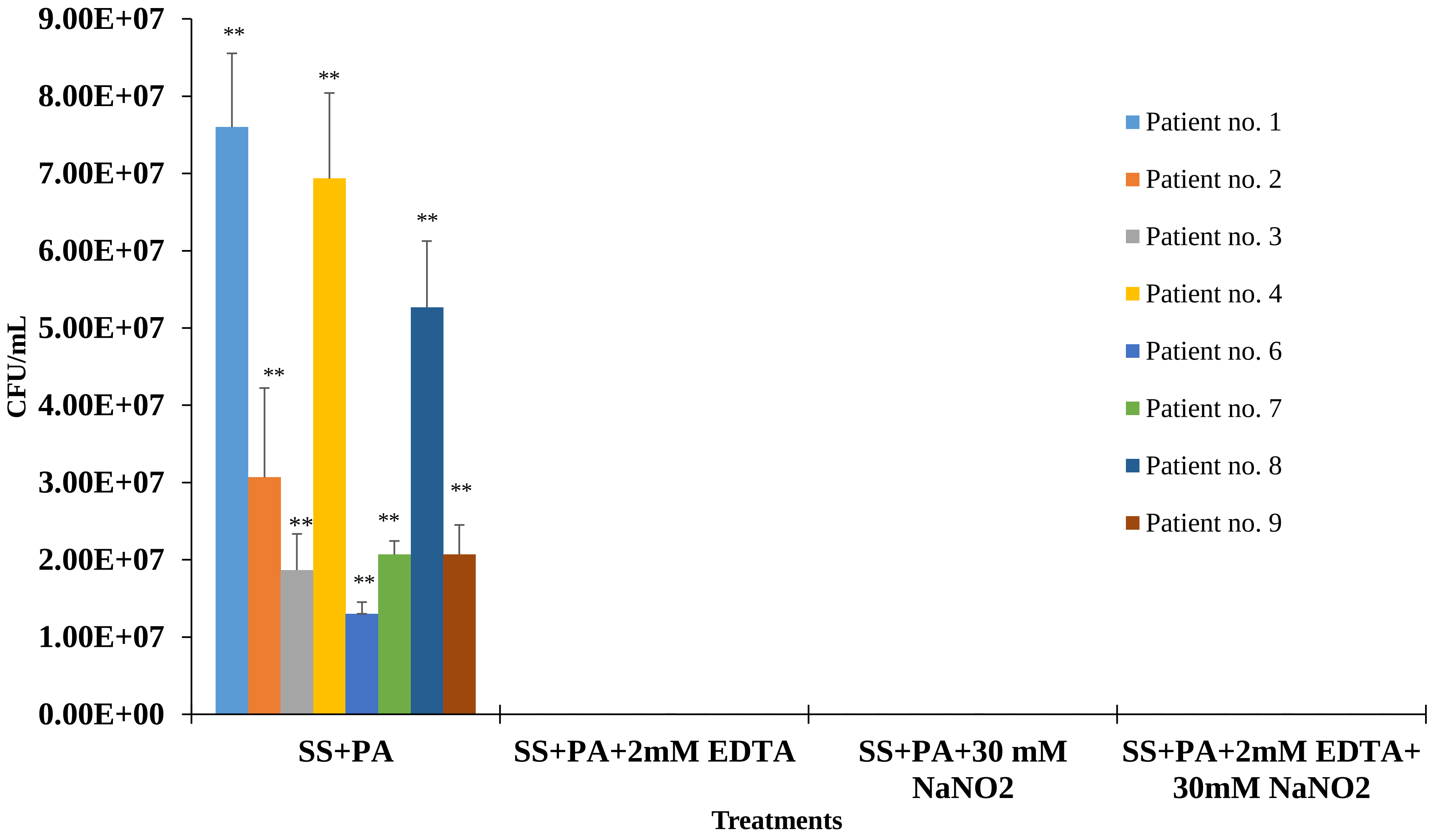

9.00E+07
8.00E+07
7.00E+07
6.00E+07
5.00E+07
4.00E+07
3.00E+07
2.00E+07
1.00E+07
0.00E+00
CFU/mL
**
Patient no. 1
Patient no. 2
Patient no. 3
Patient no. 4
Patient no. 6
Patient no. 7
Patient no. 8
Patient no. 9
SS+PA
SS+PA+2mM EDTA
SS+PA+30 mM NaNO2
SS+PA+2mM EDTA+ 30mM NaNO2
Treatments

Barry et al., 2021.
Fig. S2.

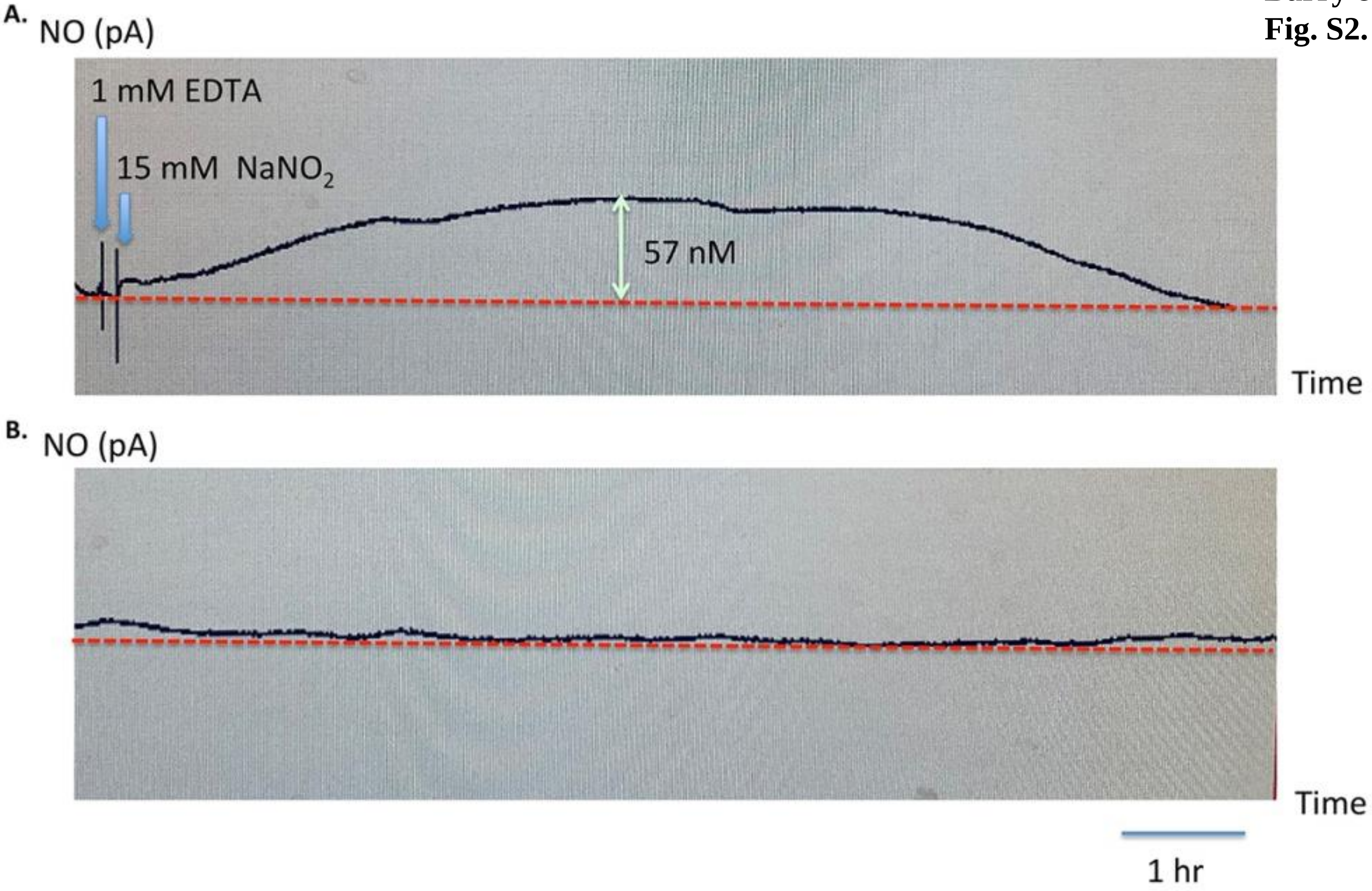

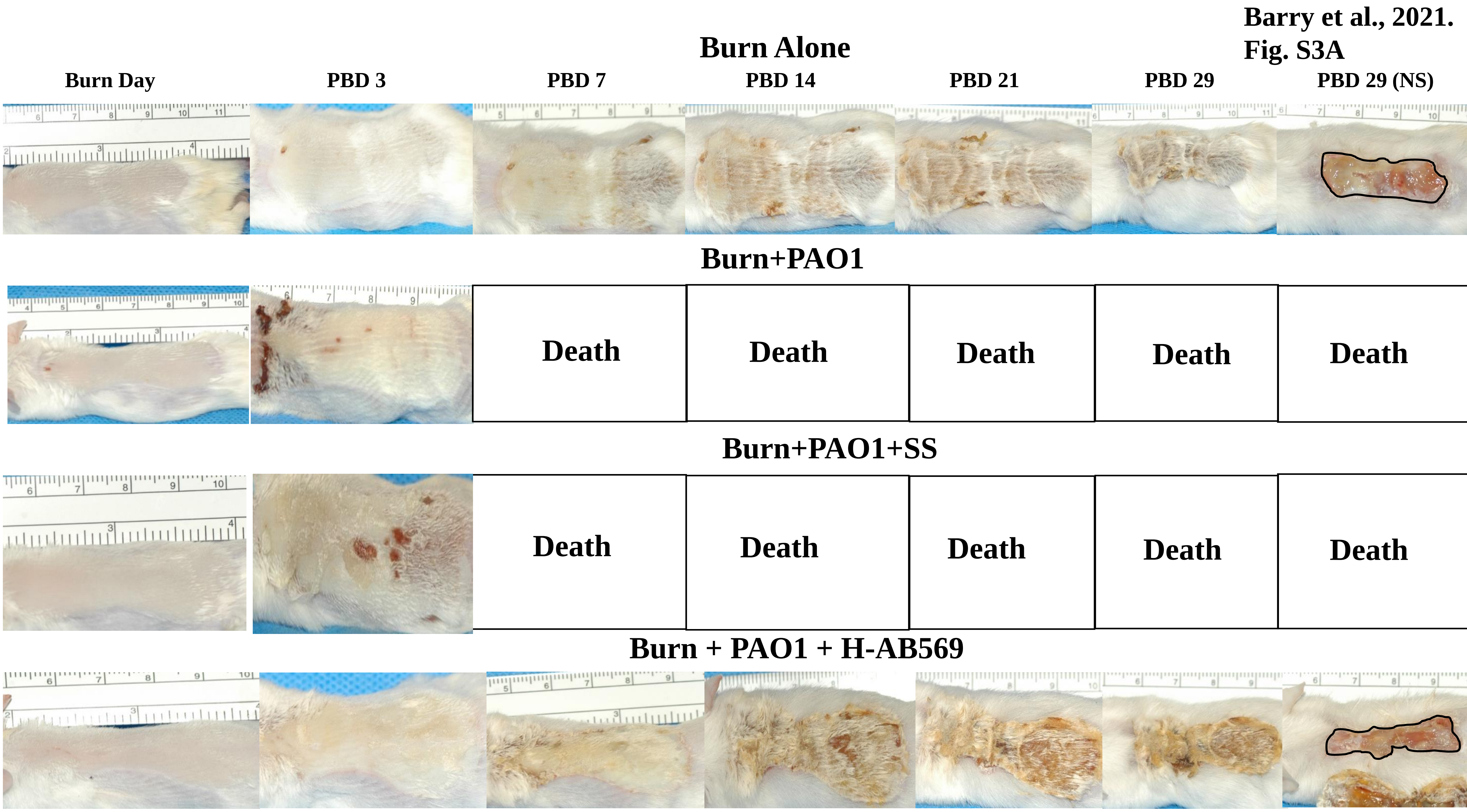
Barry et al., 2021.
Fig. S3A
Burn Alone
Burn Day
PBD 3
PBD 7
PBD 14
PBD 21
PBD 29
PBD 29 (NS)
Burn+PAO1
Death
Death
Death
Death
Death
Burn+PAO1+SS
Death
Death
Death
Death
Death
Burn + PAO1 + H-AB569

Barry et al., 2021.
Fig. S3B.

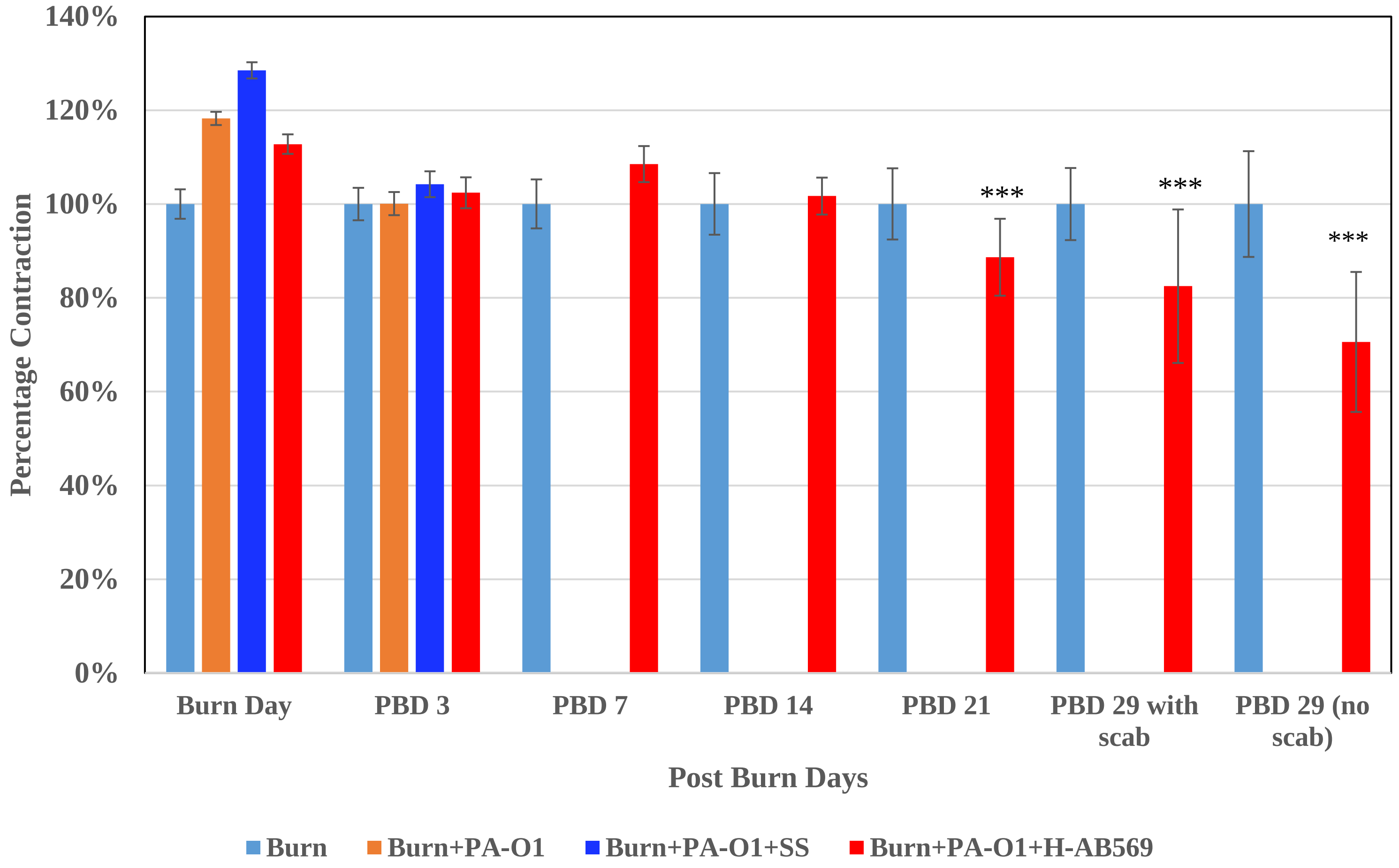

Barry et al., 2021.
Fig. S4.

A

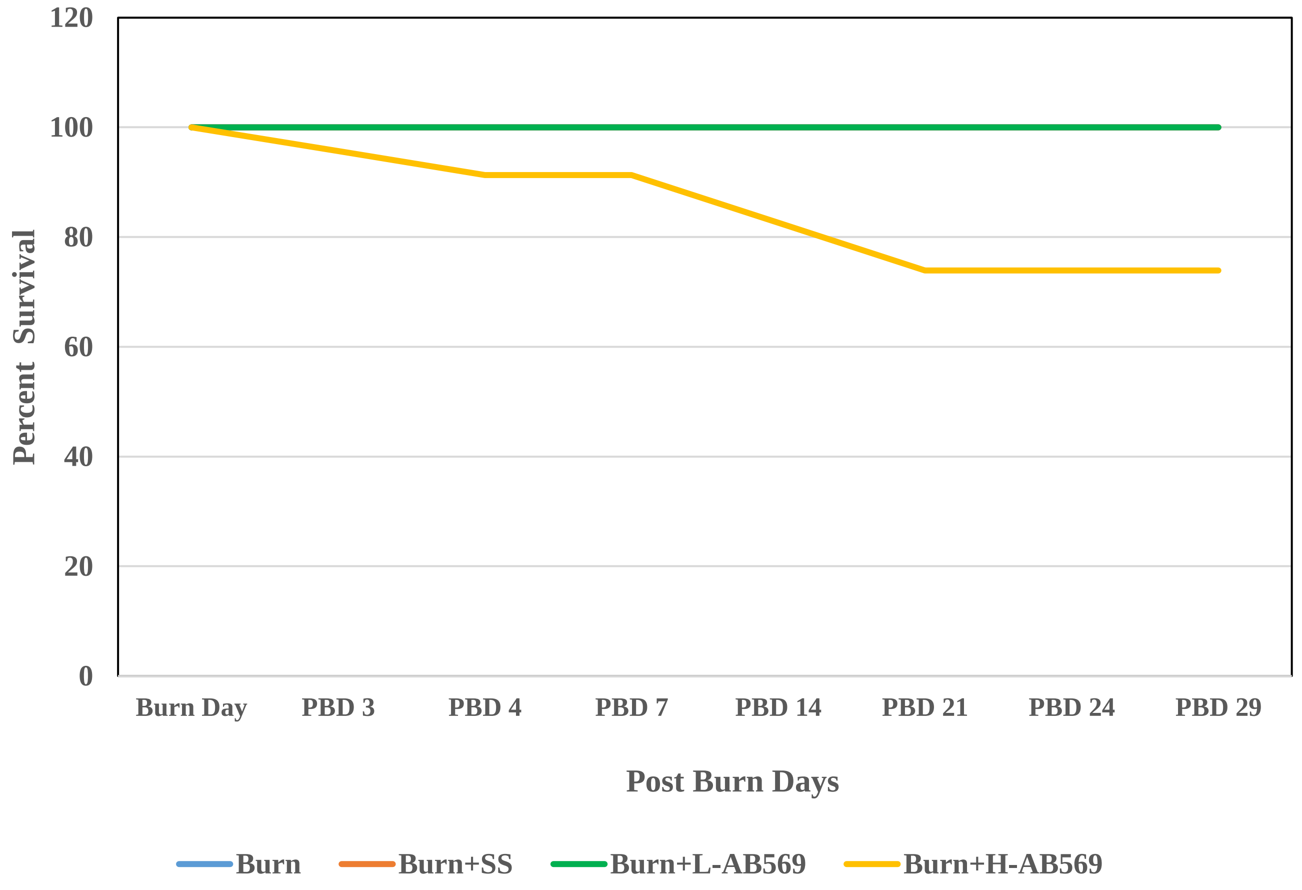

Barry et al., 2021.
Fig. S5.

A

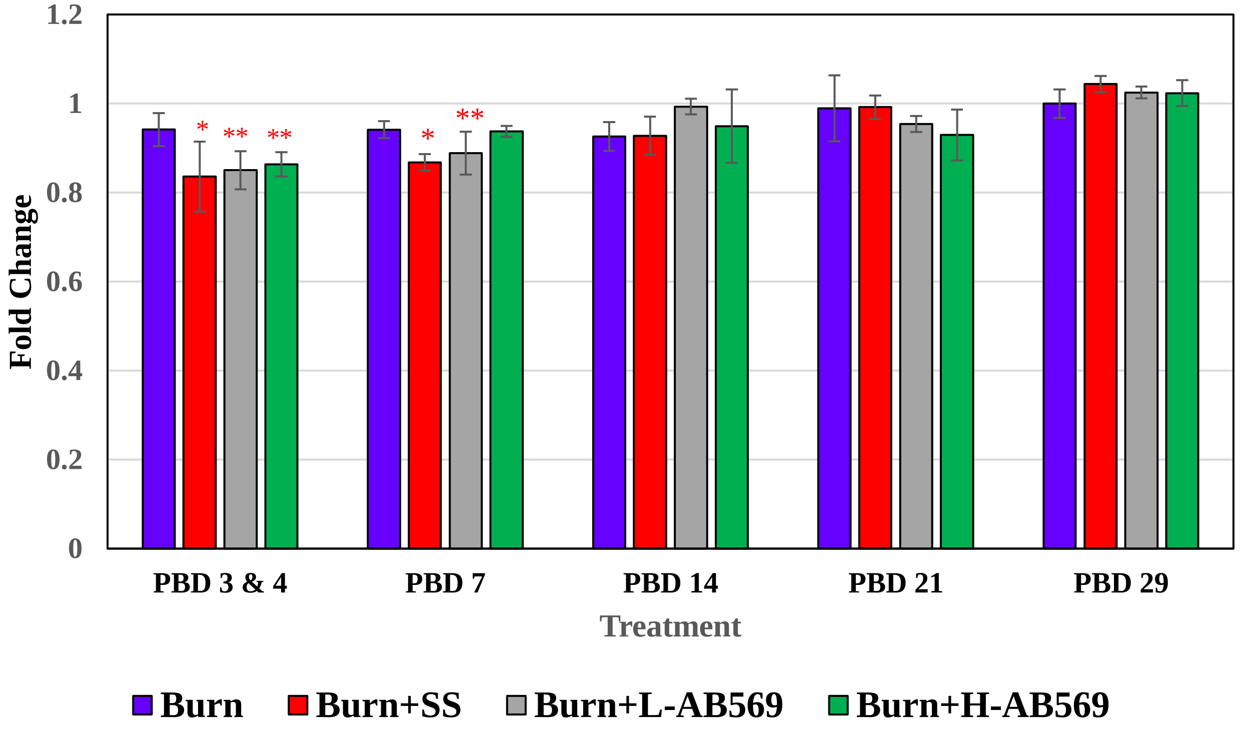

B

Barry et al., 2021.
Fig. S5.

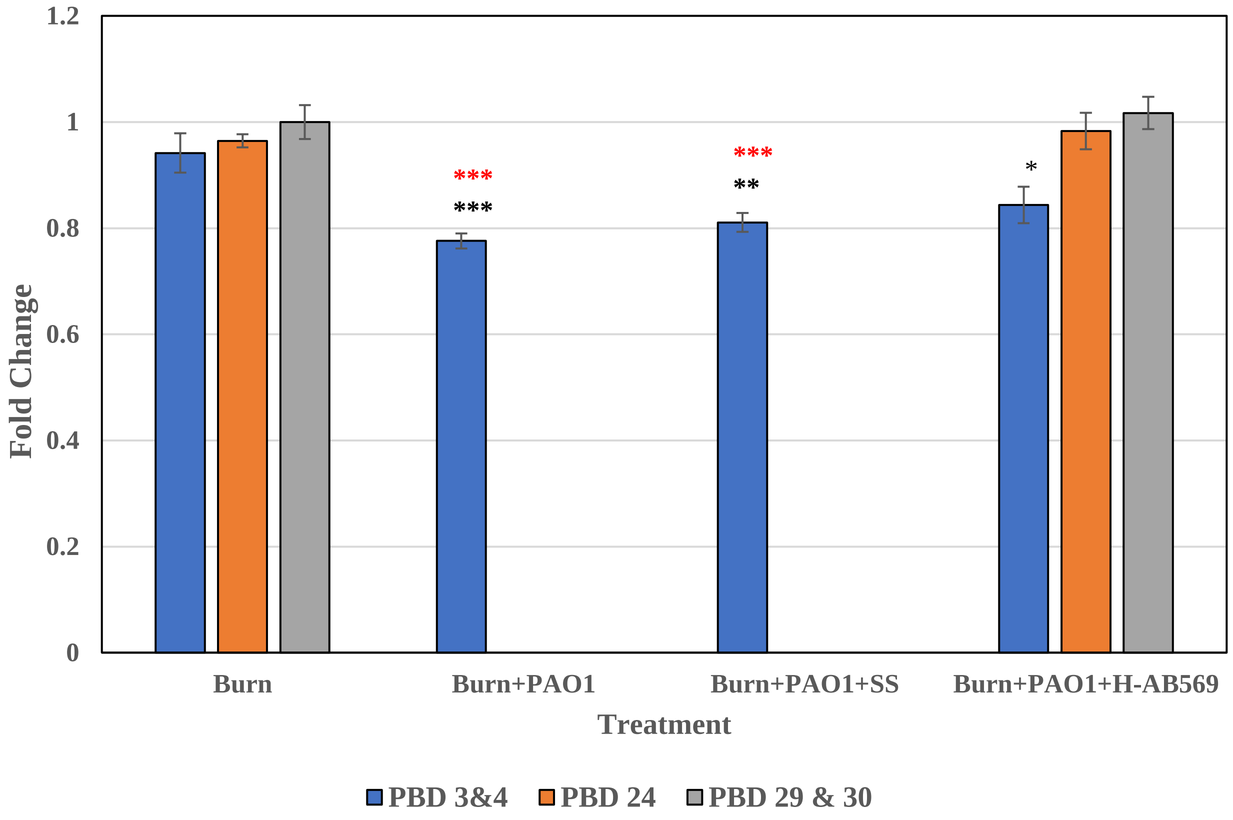

Barry et al., 2021.
Fig. S6.

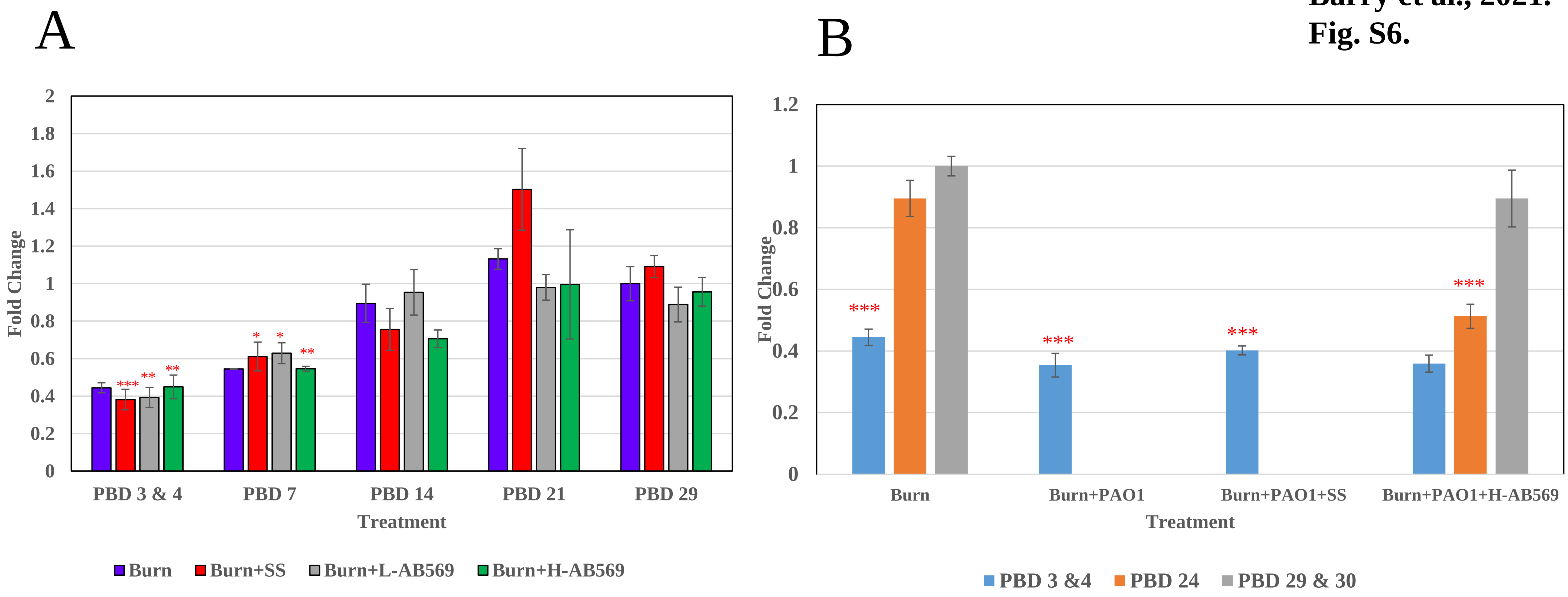

Barry et al., 2021.
Fig. S6.

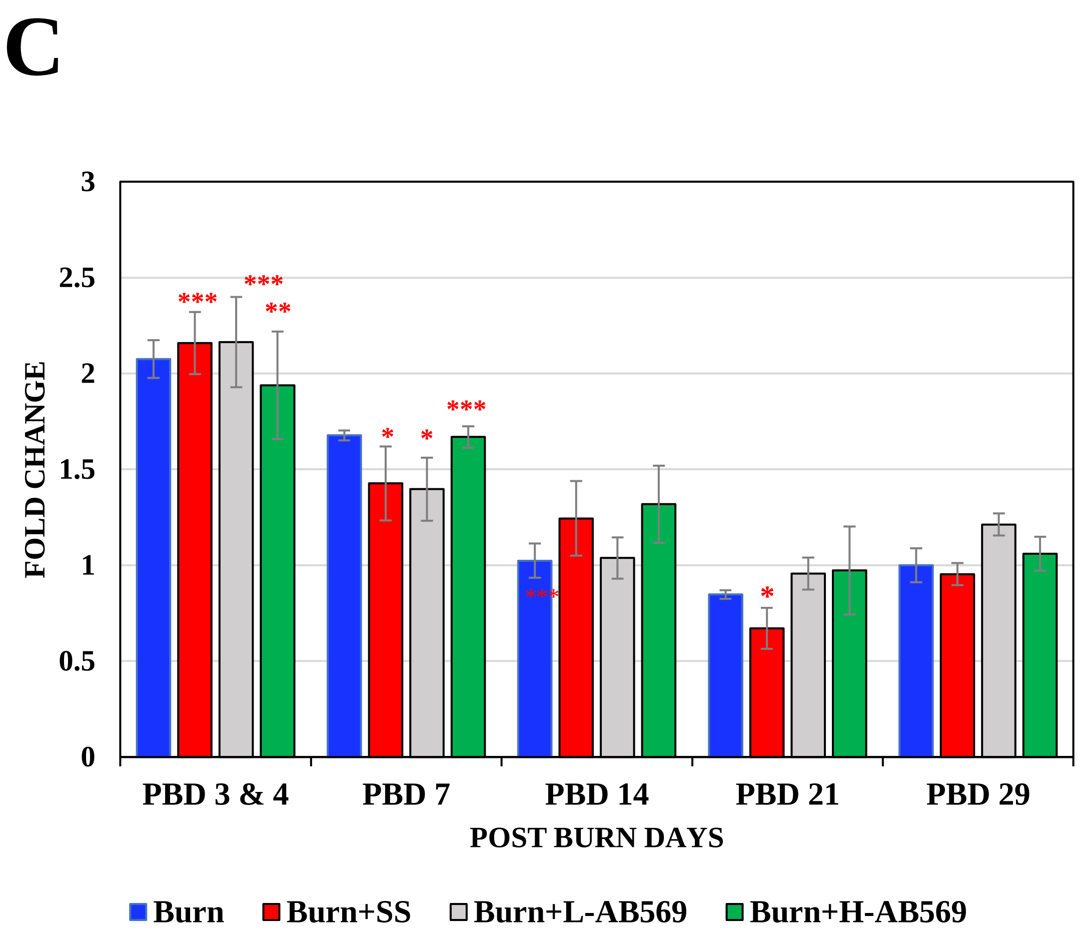

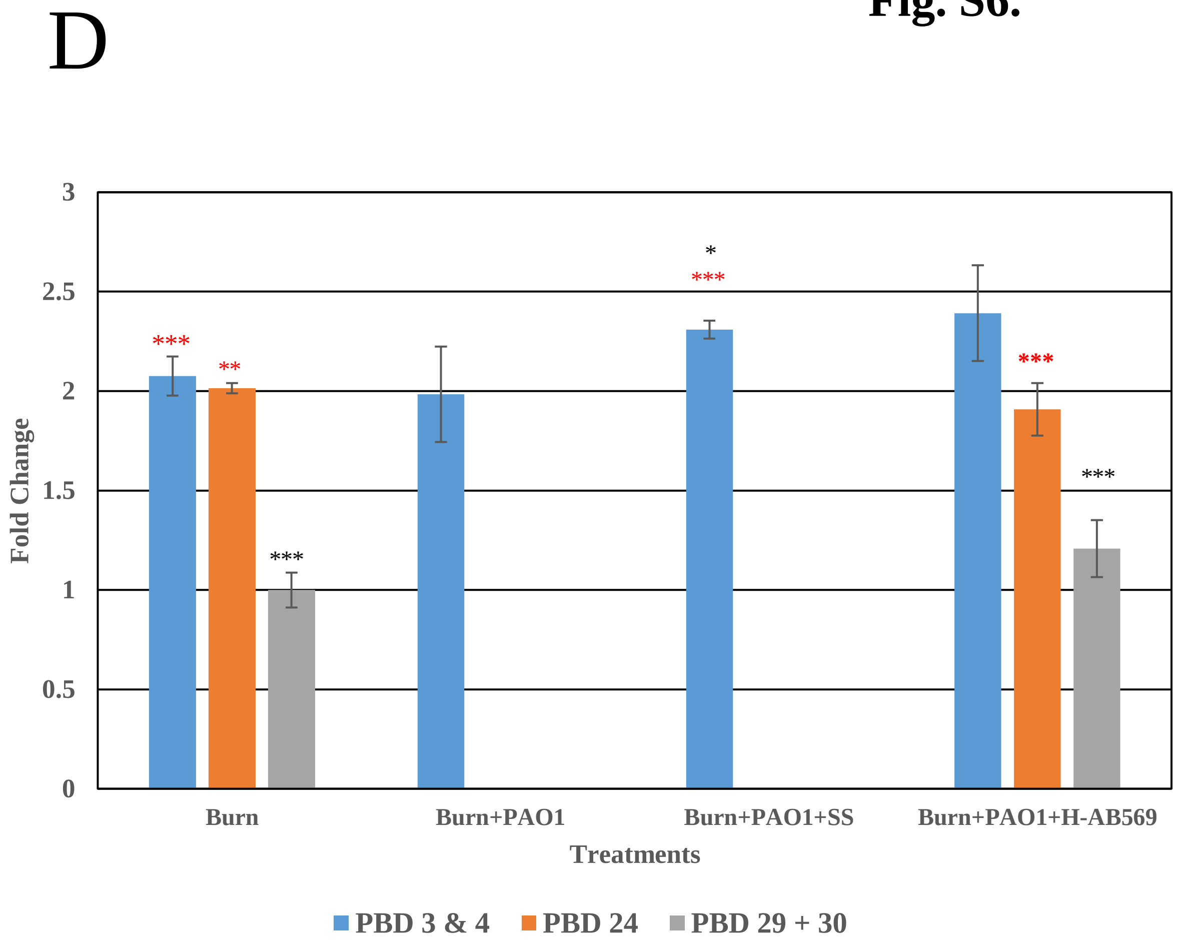

Supplement: Supplemental file 1 — Supplemental material. Download IAI.00336-21-s0001.pdf, PDF file, 0.5 MB [file iai.00336-21-s0001.pdf]
