# Supplementary material for: AB569, a Novel, Topical Bactericidal Gel Formulation, Kills Pseudomonas aeruginosa and Promotes Wound Healing in a Murine Model of Burn Wound Infection
Source: Infect Immun. 2021 Oct 15;89(11):e00336-21. doi: 10.1128/IAI.00336-21 (PMC8519293; doi:10.1128/IAI.00336-21)
Supplement: Supplemental file 2 — Supplemental material. Download IAI.00336-21-s0002.pdf, PDF file, 0.2 MB [file iai.00336-21-s0002.pdf]

Supplemental Tables

Table S1. Clinical strains of PA isolated from burn wounds (n=21) and PAO1 subjected to AB569 gel sensitivity testing using Checkerboard assay showed that all of the clinical strains of *PA* and PAO1 exhibited week synergy and overall AB569 efficiently killed all the *PA* tested. FICI- Fractional Inhibitory Concentration Index. FICI <0.05 is synergistic; 0.5<FICI<1 is weak synergistic.

Strains	MIC_{EDTA} (mM)	MIC_{NaNO2} (mM)	FIC_{EDTA} (mM)	FIC_{NaNO2} (mM)	FIC Index (FICI)	
PAO1	0.833±0.289	18.667±12.220	0.333±0.144	4.083±3.876	0.594±0.136	Weak Synergy
Burn #1-1	0.5±0	26.667±9.238	0.25±0	5.333±2.309	0.708±0.072	Weak Synergy
Burn #1	0.833±0.289	18.667±12.220	0.417±0.144	4.167±3.752	0.604±0.130	Weak Synergy
Burn #2-1	0.833±0.289	26.667±9.238	0.333±0.144	6.667±2.309	0.667±0.144	Weak Synergy
Burn #2	0.5±0	14.667±15.144	0.188±0.108	7.333±7.572	0.875±0.217	Weak Synergy
Burn #3-1	0.667±0.289	21.333±9.238	0.208±0.072	5.333±2.309	0.583±0.144	Weak Synergy
Burn #3	0.833±0.289	24±13.856	0.333±0.144	6.667±2.309	0.667±0.289	Weak Synergy
Burn #4-1	0.5±0	21.333±9.238	0.25±0	9.333±6.110	0.833±0.144	Weak Synergy
Burn #4	0.667±0.289	13.333±4.618	0.125±0.108	2.75±2.165	0.677±0.126	Weak Synergy
Burn #5-1	0.5±0	21.333±9.238	0.167±0.072	2.667±1.155	0.625±0	Weak Synergy
Burn #5	0.5±0	13.333±4.618	0.25±0	4.667±3.055	0.584±0.191	Weak Synergy
Burn #6-1	0.5±0	13.333±4.618	0.167±0.072	2.333±1.523	0.521±0.036	Weak Synergy
Burn #6	0.667±0.289	18.667±12.220	0.25±0	5.333±2.309	0.75±0	Weak Synergy
Burn #7-1	0.5±0	21.333±9.238	0.19±0.09	6±3.464	0.667±0.144	Weak Synergy
Burn #7	0.833±0.289	24±13.856	0.25±0	6.667±2.309	0.667±0.289	Weak Synergy
Burn #8-1	0.5±0	21.333±9.238	0.25±0	0.75±0.433	0.537±0.023	Weak Synergy
Burn #8	0.667±0.289	16±0	0.333±0.144	2.167±1.756	0.635±0.109	Weak Synergy
Burn #9-1	0.5±0	21.333±9.238	0.167±0.072	4.666±3.055	0.541±0.191	Weak Synergy
Burn #9	0.5±0	13.333±4.618	0.167±0.072	5.333±2.309	0.667±0.144	Weak Synergy
Burn #10-1	0.416±0.144	16±0	0.125±0	4±0	0.583±0.144	Weak Synergy
Burn #11-1	0.667±0.289	32±27.713	0.167±0.072	8±0	0.667±0.315	Weak Synergy
Burn #12-1	0.5±0	26.667±9.238	0.167±0.072	6±3.464	0.604±0.130	Weak Synergy

Supplemental Figures

Fig. S1. *In vitro* studies showing the efficacy of AB569 in killing bioluminescent PAO1 (XEN 41) and clinical strains of PA. (**A**) Broth based killing assay showing XEN41 killing is complete and maintained 48 hr after treatment with 30 mM A-NO_2^- (acidified nitrite) and 2 mM EDTA and 30 mM A-NO_2^-. (**B**) Representative IVIS® images of PAO1XEN41 bioluminescence in cultures treated with 30 mM A-NO_2^- or EDTA (2 mM); and 30 mM A-NO_2^- and 2 mM EDTA. Solosite in LB pH 6.5, and untreated *PA* XEN41 were used as negative and positive control respectively. Cultures were grown in LB media with a pH of 6.5 along with 1% Solosite gel used as a vehicle. Tube 1; LB 6.5+ SS, Tube 2; LB 6.5+ SS+ PAO1 Xen 41, Tube 3; LB pH 6.5+SS + PAO1Xen41 +2 mM EDTA, Tube 4; LB 6.5+SS+ PAO1 Xen41 +30 mM A-NO_2^-, Tube 5; LB 6.5+SS+ PAO1 XEN41 + 2 mM EDTA+30 mM A-NO_2^-. (**C**) Clinical strains of *PA* isolated from 8 different burn patients were subjected to broth based killing assay. All the experiments were performed independently for n=3 in triplicate. Student's t test was used to determine the statistical significance. $p<0.05$ was considered statistically significant. SS; Solosite.

Fig. S2. Amperometric detection of NO generation by an AB569 SS gel formulation. (**A**) with AB569; (B) without AB569. pA = amperage unit to detect NO levels.

Fig. S3. AB569 enhances wound closure in infected burn wounds. (**A**) Gross images of the wounds were captured, and analysis of wound closure was performed using NIH Image J. Wounds were photographed weekly using a standard digital camera. (**B**) Rate of wound closure in uninfected treated burn wounds measured by Image J from burn day to PBD 29. The data is normalized to burn for each post burn day. The student's t-test compared the black (*) p-values to burn day and the red (*) p-values to high AB569, on respective days. (Burn: n= 20; H-AB569: n= 23; PAO1+SS: n=4; PAO1: n=10; PAO1+H-AB569: n=9). NS; No Scab.

Fig. S4. Survival rates of uninfected animals was not altered after AB569 treatment. (**A**) The survival rates of uninfected burn wounds from Burn day to PBD 29 in all the treatment groups (Total no. of animals; n =87).

Fig. S5. Body weight is significantly improved in both infected and uninfected burn wounds. **(A)** Body weight (uninfected wounds). After normalizing the data to Burn PBD 29, a student's t-test compared PBD 29 (red *) body weight to the uninfected burn wounds and treatments. (Burn PBD 3: n=5; Burn PBD 7: n=3; Burn PBD 14: n=3; Burn PBD 21: n=2; Burn PBD 29: n= 5; H-AB569 PBD 3& 4: n= 10; H-AB569 PBD 7: n= 3; H-AB569 PBD 14: n= 2; H-AB569 PBD 21: n=2; H-AB569 PBD 29: n= 7; SS PBD 3: n= 3; SS PBD 7: n= 3; SS PBD 14: n= 3; SS PBD 21: n= 3; SS PBD 29: n= 10; L-AB569 PBD 3: n= 3; L-AB569 PBD 7: n= 3; L-AB569 PBD 14: n= 3; L-AB569 PBD 21: n= 3; L-AB569 PBD 29: n= 8). **(B)** Body weight (infected wounds). After normalizing the data to PBD 29, a student's t-test compared PBD 29 (red *) body weight to the infected burn wounds and treatments. (Burn: n=5; PAO1 + H-AB569 PBD 3 & 29: n= 10; PAO1 + H-AB569 PBD 24: n= 6; PA: n= 6; *PA* + SS: n= 4). $p<0.05$ was considered statistically significant. *$p<0.05$; **$p<0.01$ ***$p<0.001$. PBD;. SS; Solosite, L-AB569; Low AB569, H-AB569; High AB569.

Fig. S6. AB569 influences the changes in spleen weight contributing to the survival of animals. **(A)** After normalizing the data to Burn PBD 29, a student's t-test compared Burn alone PBD 29 (red *) spleen weight to the uninfected burn wounds and treatments. (Burn alone PBD 3: n=5; Burn alone PBD 7: n=3; Burn alone PBD 14: n=3; Burn alone PBD 21: n=2; Burn alone PBD 29: n= 5; H-AB569 PBD 3& 4: n= 10; H-AB569 PBD 7: n= 3; H- AB569 PBD 14: n= 2; H-AB569 PBD 21: n=2; H-AB569 PBD 29: n= 7; SS PBD 3: n= 3; SS PBD 7: n= 3; SS PBD 14: n= 3; SS PBD 21: n= 3; SS PBD 29: n= 10; L-AB569 PBD 3: n= 3; L-AB569 PBD 7: n= 3; L- AB569 PBD 14: n= 3; L-AB569 PBD 21: n= 3; L-AB569 PBD 29: n= 8).

(B) After normalizing the data to Burn PBD 29, a student's t-test compared Burn PBD 29 (red *) spleen weight to the infected burn wounds and treatments. (Burn alone : n=5; PAO1 + H-AB569 PBD 3 & 29: n= 10; PAO1 + H-AB569 PBD 24: n= 6; PAO1: n= 6; PAO1 + SS: n= 4).

(C) After normalizing the data to Burn PBD 29, a student's t-test compared PBD 29 (red *) body to spleen ratio to the uninfected burn wounds and treatments. (Burn alone PBD 3: n=5; Burn alone PBD 7: n=3; Burn alone PBD 14: n=3; Burn alone PBD 21: n=2; Burn alone PBD 29: n= 5; High AB569 PBD 3& 4: n= 10; High AB569 PBD 7: n= 3; High AB569 PBD 14: n= 2; H- AB569 PBD 21: n=2; H-AB569 PBD 29: n= 7; SS PBD 3: n= 3; SS PBD 7: n= 3; SS PBD 14: n= 3; SS PBD 21: n= 3; SS PBD 29: n= 10; L-AB569 PBD 3: n= 3; L-AB569 PBD 7: n= 3; L-AB569 PBD 14: n= 3; L-AB569 PBD 21: n= 3; L-AB569 PBD 29: n= 8). **(D)** After normalizing the

data to Burn PB 29, a student's t-test compared Burn PBD 29 (red *) body to spleen ratio to the infected burn wounds and treatments. (Burn alone : n=5; PAO1 + H-AB569 PBD 3 & 29, n= 10; *PA-01* + H-AB569 PBD 24: n= 6; PAO1, n= 6; PAO1 + SS, n= 4). PBD; post-burn day. $p<0.05$ was considered statistically significant. *$p<0.05$; ***$p<0.001$. PBD; post-burn day, SS; Solosite, L-AB569; Low AB569, H-AB569; High AB569.
